# Gut microbes–spinal connection is required for itch sensation

**DOI:** 10.1080/19490976.2025.2495859

**Published:** 2025-04-27

**Authors:** Tong Jin, Si-Yuan Li, Hong-Li Zheng, Xiao-Dan Liu, Yue Huang, Gan Ma, Ya-Xuan Zhao, Xiao-Tian Zhao, Li Yang, Qi-Hui Wang, Hong-Jun Wang, Chengyong Gu, Zhiqiang Pan, Fuqing Lin

**Affiliations:** a Jiangsu Province Key Laboratory of Anesthesiology, Jiangsu Province Key Laboratory of Anesthesia and Analgesia Application Technology, NMPA Key Laboratory for Research and Evaluation of Narcotic and Psychotropic Drugs, Xuzhou Medical University, Xuzhou, China; b Department of Pain, Shanghai Tenth People’s Hospital, School of Medicine, Tongji University, Shanghai, China; c Anesthesiology Department, Suzhou Municipal Hospital, Nanjing Medical University Affiliated Suzhou Hospital, Suzhou, China; d Department of Anesthesiology, The Sixth Affiliated Hospital of Guangzhou Medical University, Qingyuan, China

**Keywords:** Gut microbe, itch, FTO, RNA m^6^A, *B. fragilis*, acetyl-l-carnitine

## Abstract

The gut microbiota has been linked to a number of neurological disorders. However, it is unclear whether the gut microbiota is involved in the genesis of chronic itch, a refractory condition that afflicts patients both physically and mentally. Here, we report that depletion of gut microbiota enhances tolerance to itch in mice orally administered with antibiotics (ABX) and mice free of germ. Of note, oral gavage with *Bacteroides fragilis* (*B. fragilis*), a prominent species of the genus *Bacteroides* with most differential change, corrected the ABX-induced itch dysfunction through its driven metabolite acetyl-l-carnitine (ALC). Mechanistically, gut microbiota or *B. fragilis* depletion caused a decrease in RNA N6-methyladenosine (m^6^A) demethylase FTO expression in the dorsal horn and a consequent increase in RNA m^6^A sites in Mas-related G protein-coupled receptor F (*MrgprF*) mRNA, leading to decreased MRGPRF protein. The downregulation of FTO was triggered by inactivation of ETS proto-oncogene 1 (ETS1), a transcription factor that binds to the *Fto* promoter. These findings support a gut microbe – spinal connection in modulation of itch sensation in RNA m^6^A epigenetic-dependent manner and highlight a critical role of ALC in linking the altered *B. fragilis* and itch dysfunction.

## Introduction

Chronic itch is a debilitating disorder which is characterized by the desire to scratch.^1^ It is estimated that 10%–20% of the population suffer from chronic itch at some point in their lifetime.^2^ Chronic itch is initiated by irritation of the skin or mucous membrane, as well as by systemic conditions including kidney failure, cirrhosis, dermatitis, herpes zoster, and liver cancer.^3^ Increasing evidence showed that gut microbiota-derived metabolites can potentially modulate the function of central nervous system (CNS) and subsequent behavior through gut-CNS (such as gut-spinal cord or gut-brain) axis.^4^ For instance, fecal transplantation increases the level of circulating short-chain fatty butyrate, changes expression of genes involved in lipid metabolism and calcium handling in cells of the nerve system, resulting in the improvement of neuropathic pain in obese mice.^5^ Although a variety of factors eliciting itch behavior have been observed clinically, the understanding of the development of chronic itch is still in its infancy, owing to the complexity of the underlying systems.

The gut microbiota – the population of microorganisms colonizing the gastrointestinal tract – serves as an important regulator for host intestinal homeostasis and has a crucial role in the human body: for example, it is linked to the development and normal functioning of the body’s immune and nervous systems. It is reported that oral gavage of the human commensal *Bacteroides fragilis* (*B. fragilis*) of *Bacteroidota* phylum modulates levels of several metabolites, attenuates gut permeability, leading to the amelioration of the defects in communicative, stereotypic, anxiety-like and sensorimotor behaviors in a mouse model of autism spectrum disorder. The alterations in the gut microbiota are increasingly implicated in neurological disorders, including neuroinflammation,^6^ multiple sclerosis,^7^ autism spectrum disorder,^8^ Parkinson’s disease,^9^ amyotrophic lateral sclerosis (ALS),^10^ cognitive dysfunction,^11^ anxiety-like behavior,^12^ depression,^13^ and nociception.^14^ However, whether and how the gut microbiota regulates itch sensation remains unknown.

Epigenetics is the study of how cells regulate gene activity without altering the DNA sequence, through various modifications such as DNA or RNA methylation, histone acetylation, and others. RNA N6-methyladenosine (m^6^A) methylation is one of the most abundant types of internal mRNA modification, and present in over 25% of mammalian mRNA. RNA m^6^A constitute a major discovery in the field of epigenetic modification, secondly to DNA and histone methylation.^15^ m^6^A modification is a reversible process that is dynamically “written” by the METTL3/METTL14/WTAP methyltransferase complex and erased by the demethylases fat-mass and obesity-associated protein (FTO) or alkB homolog 5 (ALKBH5). Many studies have established the connection between m^6^A and mRNA splicing in the nucleus,^15^ along with its involvement in degradation,^16^ and translation efficiency.^17^ Furthermore, RNA m^6^A has been found to be associated with adult neurogenesis,^18^ synaptic formation,^19^ memory formation and consolidation,^20^ as well as the processes related to peripheral nerve injury and inflammatory pain,^21^ and stroke.^22^ Mice lacking FTO in sensory neurons of the dorsal root ganglia (DRG)^23^ or in thalamic neurons^22^ have markedly attenuated pain-like behavior after peripheral nerve injury or hemorrhage. Consequently, FTO-dependent m^6^A likely plays a critical role in pathophysiology in the mammalian central nervous system. Yet, whether FTO also participates in the genesis of itch is not known.

Here we determined the effects of gut microbiota on itch sensation using antibiotics-deleted gut microbiota (ABX) and germ-free mice. The chloroquine-evoked itch threshold, locomotor, microbiota profiles, m^6^A-related proteins and its upstream/downstream targets in spinal dorsal horn were examined, as were measurements of neuropathic itch signals. Furthermore, to establish a causal association between gut microbiota and itch, the potential role of *B. fragilis* and its derived acetyl-l-carnitine (ALC) on itch alterations was assessed in ABX mice. We found that deletion of the gut microbiota caused enhanced tolerance of itch in mice as well as dramatic decreases in FTO and Mas-related G protein-coupled receptor F (MRGPRF), a member of itch-related gene family, in the dorsal horn of the spinal cord. Oral gavage of *B. fragilis* or the intraperitoneal injection of *B. fragilis*-derived acetyl-l-carnitine, in part, ameliorates itch dysfunction induced by depletion of gut microbiota. Therefore, we demonstrated that the gut microbiota is a key initiator of chronic itch via its effects on FTO-dependent m^6^A modification of MRGPRF expression.

## Materials and methods

### Mice and the itch behavior test

Two groups of adult animals consisting of BALB/C mice (from Xuzhou Medical University animal facility) and germ free (GF) mice (from Gempharmatech Co., Ltd, China) were used in this study and were housed 4–6 per cage with a 12-h light/dark cycle and ad libitum access to rodent chow and water. Mice used for experiments were 8-week-old males (20–30 g). All GF animals were housed in Sterile isolator (bse-101, Shandong shinva Medical Equipment Co., LTD). When required, animals were shaved using a small electric shaver 1 day prior to recording. To assess chloroquine-evoked itch behaviors, mice received a subcutaneous injection of chloroquine (200 μg in 50 μl, dissolved in PBS) into the neck. Mice were videotaped for 60 min following injection. The amount of time each mouse spent scratching and the number of scratch bouts were quantified for each minute. One bout of scratching was defined as an episode in which a mouse lifted its paw and scratched continuously for any length of time, until the paw was returned to the floor.

### Antibiotic treatment

According to the previous described^24^ with minor modification, mice were received with a combination of broad-spectrum antibiotics in their drinking water containing neomycin (1 mg/mL), ampicillin (1 mg/mL), metronidazole (1 mg/mL), and vancomycin (0.5 mg/mL) for 7 days, during this period, the antibiotics drinking water was renewed three times, and then switched to normal drinking water to eliminate the pharmacological effect of antibiotics. For mock treatment, mice were received with normal drinking water.

### Fecal microbiome transplantation

The fecal microbiome transplantation experiment was carried out as the previous described^24,25^ with minor modification. Fecal samples were freshly collected from donor mice using the tail-lifting method, where the mouse’s tail was lifted, and the lower abdomen was gently pressed with a finger to induce defecation. and suspended at 10 mg/ml in sterile PBS, after thoroughgoing vibration, filtered through a pore size of 70 μm, filtering liquor was centrifuged at 4 °C for 5 min and continued to take suspension centrifugal washing to gain the final bacterial suspension, which was diluted with an equal volume of sterile PBS (approximately containing 10^8^ CFU/ml fora). Antibiotic-treated mice were colonized by oral gavage of 100 ul suspension. For mock treatment, mice were gavaged with sterile PBS. Mice were housed in microisolator cages and handled aseptically.

### 16S rDNA sequencing of gut microbiota

Fecal pellets were collected from fresh feces mouse using tail-lifting method mentioned above. Microbial DNAs of fecal was extracted using a Stool DNA extraction kit (OMEGA Stool DNA Kit) according to the kit instruction and previous reports.^26^ Construction of library, sequencing and informatics analysis were finished by LC-Bio Technologies (Hangzhou) Co., Ltd. Briefly, a conserved PCR primer targeting variable region in gut microbiota 16S/ITS2 rDNA gene were used in the amplification, followed by the addition of sequencing adapters and barcodes for amplification. After agarose gel electrophoresis, the target fragments were recovered and purified, respectively with an AxyPrep PCR Cleanup Kit and a Quant-iT PicoGreen dsDNA Assay Kit. The quantified library was sequenced with an Illumina Novaseq platform with a paired-end sequencing protocol (2  ×  250 bp). A bioinformatics pipeline was used to analyze the species diversity of gut microbiota after paired-end reads (FLASH, v1.2.8 for 16S) and high-quality clean tags (fqtrim, v0.94; Chimeric sequences filtering Vsearch software v2.3.4). QIIME 2 was applied to analyze the Alpha diversity and beta diversity, and R v3.5.2 to obtain the corresponding images. Sequence alignment and species annotation was carried out through SILVA and NT-16S assay. Bacteria taxonomy was shown via the relative abundance (X bacteria count/total count).

### Metagenomics sequencing of gut microbiota

The microbial DNAs from mouse gut were separated using the OMEGA Stool DNA Extraction Kit. Library construction, sequencing, and informatics analysis were performed by LC-Bio Technologies (Hangzhou) Co., Ltd. After fragmenting the DNA with dsDNA Fragmentase (M0348S, NEB), the DNA library was constructed using the Fast DNA Library Prep Set for Illumina (CW3045M), and the sequencing was conducted in the Illumina Novaseq platform. Raw sequencing reads were filtered, adapters were removed using Cutadapt v1.9, and low-quality reads were trimmed with Fqtrim v0.94. The reads were aligned to the host genome to eliminate host contamination using Bowtie2 v2.2.0. Quality-filtered reads were then de novo assembled to construct the metagenome for each sample using MEGAHIT v1.2.9. All coding regions (CDS) of the metagenomic contigs were predicted with MetaGeneMark v3.26. The CDS sequences from all samples were clustered using CD-HIT v4.6.1 to obtain unigenes. The abundance of unigenes in each sample was estimated using TPM based on the number of aligned reads from Bowtie2 v2.2.0. The lowest common ancestor taxonomy was determined by aligning the protein sequences against the NCBI non-redundant protein database (NR) using DIAMOND v0.9.14, in which the classification follows the NCBI Taxonomy. For sequences with ambiguous or incomplete taxonomic information, the lowest reliable taxonomic level was assigned based on the alignment results.

### Bacterial strains and gavage

The wild-type *Bacteroides fragilis* strain (24309) was provided by the China Center of Industrial Culture Collection (CICC). *Bacteroides fragilis* were freshly cultured in anaerobic conditions as described method,^24^ and then washed, pelleted and resuspended at 5Х10^9^ cfu/ml in prereduced PBS. Mice were gavaged every 12 hours with 200 ul bacterial suspension as the previous described^25^ with minor modifications.

### Locomotor function

Locomotor function of three reflex testing including placing, grasping, and righting were examined after itch tests as the previous described.^27^ Briefly, 1) Placing reflex: The hind limbs were positioned slightly lower than the forelimbs, with the dorsal surfaces of the hind paws in contact with the edge of the table. It was recorded whether the hind paws were reflexively placed on the table surface; 2) Grasping reflex: Following the placement of the animal on a wire grid, we documented whether the hind paws grasped the wire on contact; 3) Righting reflex: After placing the animal on its back on a flat surface, we noted whether it immediately returned to a normal upright position. Each trial was conducted five times with five-minute intervals, and the scores for each reflex were recorded based on the number of times the normal reflex occurred.

### Spinal tissue collection

Mice were anesthetized with 10% pentobarbital sodium, and the spinal cord corresponding to the lumbar segments (L3–L5) was removed rapidly. The spinal cord was separated and snap frozen in liquid nitrogen, then stored at  −  80°C.

### Cell line culture and transfection

As the previous method, HEK293T cells or HT-22 cells were cultured with Dulbecco’s Modified Eagle’s Medium (DMEM; Gibco/ThermoFisher Scientific, Waltham, MA) containing 10% v/v fetal bovine serum (FBS; Gibco/ThermoFisher Scientific) at 37°C. Lipofectamine 6000 (C0526, Beyotime) was used to co-transfect the plasmids and siRNAs in 293T cells in the light of the manufacturer’s instructions.

### RNA extraction and qRT-PCR

RNA extraction and qRT-PCR were carried out as described previously.^28^ Briefly, Trizol assay (9190, Takara) was used to extract total RNA, cDNA was obtained through reverse transcription using (dT)_18_ or random sequence oligos and reverse transcriptase M-MLV (2 641A, Takara). All PCR primers are listed in Supplementary Table S2. The change fold of target genes was calculated via 2^−ΔΔCT^ analysis.

### m^6^A dot-blot analysis

mRNA was separated by using a Dynabeads mRNA Direct Purification Kit (61011, Ambion) from homogenized dorsal horn. 100 ng mRNA per 1 μl were applied and crosslinked to Hybond-N^+^ membrane as described previously.^29^ After washes in PBST 3 times, the membrane was blocked in 5% milk for 2 hours and incubated with primary anti-m^6^A antibody (ABE572, Millipore) at room temperature for 2 hours, then washed 3 times, and incubated with HRP-conjugated IgG antibody for 2 hours, and washed 3 times in PBST. The visualized signal was detected via an ultra-sensitive ECL chemiluminescence kit (P0018S, Beyotime). The m^6^A signal quantitation was normalized to the amount of mRNA loaded.

### Plasmid construction

Plasmid preparation was carried out as the previously described.^28^ PCDH lentivirus vector was used to construct *Fto*, *Ets1*, and *MrgprF* overexpression plasmids by using PCR primers (Supplementary Table S2) and ClonExpress® II One Step Cloning Kit (Vazyme, China). PLVTHM lentivirus vector was applied to construct the *Fto* knock-down vector by using the synthesized sh-FtoF and sh-FtoR oligos with Mlu1 and ClaI sites (Supplementary Table S2). Sanger sequencing was used to confirm these constructs.

### siRNA and virus delivery

Intrathecal injections of siRNA or virus were performed according to previously described methods.^30^ 5 μl of 20 μM siRNA for *Fto*, *Ets1*, *MrgprF*, or scrambled siRNA was injected. All siRNA sequences are reported in Supplementary Table S2. 5-μl lentivirus injections were performed daily. Knock-down via *Fto*-siRNA, *Ets1*-siRNA, *MrgprF*-siRNA, or sh-*Fto* was confirmed with qRT-PCR from samples of spinal cord. Mice receiving intrathecal injection of scrambled siRNA or empty vector were used as the control.

### RNA immunoprecipitation

RNA immunoprecipitation (RIP) was carried out as previously described.^21^ Briefly, equal weights of spinal cord tissues were washed in pre-cooled PBS buffer, followed by the digestion using nuclear isolation buffer. After homogenization, precipitated and resuspended cellular nuclei in RIP buffer, collected the supernatant after centrifugation, and separated into three fractions including Input, Negative control and IP. IP fraction was incubated with m^6^A antibody (ABE572, Millipore) overnight, and then incubated with Protein G Magnetic Beads (S1430S, NEB), and collected beads and resuspended them in RIP buffer three times, and washed one time in PBS. Co-precipitated RNA was isolated by RNA Isolater extraction reagent (R401–01, Vazyme). The m^6^A level in different regions of *MrgprF* mRNA was measured by using three pairs of PCR primers for *Mrgpr*F 3’UTR region in qRT-PCR (Supplemental Table S2).

### Western blot

Protein level was measured as described previously.^30^ In brief, 20–50 μg proteins were transferred onto nitrocellulose membranes, and incubated in the antibody: FTO antibody (1:1000; A1438, ABclonal), METTL 3 antibody (1:1000; ab195352, Abcam), ETS1 antibody (1:1000; A13302, ABclonal), MRGF antibody (1:1000; ER64002, Huabio), control Tubulin antibody (1:2000; 10094–1-AP, Proteintech) or GAPDH antibody (1:2000; 10494–1-AP, Proteintech). After wash, the membranes were incubated with secondary antibody – HRP-labeled Goat Anti-mouse IgG (1:2000; A0216, Beyotime) or Goat Anti-rabbit IgG (1:1000; A0208, Beyotime). The signal intensity of immune complexes was detected using ultra-sensitive ECL chemiluminescence kit (P0018S, Beyotime).

### Double-labeling immunofluorescence

Mice were perfused by using 4% paraformaldehyde, and then post-fixed for 4–6 h using 4% paraformaldehyde, their L3–L5 spinal cords were dissected, and dehydrated using 30% sucrose overnight, and sectioned consecutively into 20-µm slices. The slices were blocked for 1 h at room temperature and washed using PBS, and then incubated with Rabbit FTO antibody (1:200; A1438, ABclonal) or ETS1 antibody (1:1000; A13302, ABclonal) and NeuN antibody (1:200; ab104224, Abcam), or GFAP antibody (1:2000; ab53554, Abcam), or IBA1 antibody (1:200; ab5076, Abcam) at 4°C overnight for 48 h. The slices were washed in PBS, followed by incubation with fluorescent-conjugated secondary antibodies Alexa Fluor 488 donkey anti-rabbit lgG (A21206, Molecular Probes) or Alexa Fluor 546 donkey anti-mouse lgG (A10036, Molecular Probes), and then mounted onto slides with DAPI. Finally, the immunofluorescence signals were obtained by a Zeiss LSM 880 confocal microscope (Zeiss, Jena, Germany).

### Chromatin immunoprecipitation (ChIP)

ChIP assays were performed according to the instruction of the ChIP Assay Kit (P2078, Beyotime) and the described.^23^ 1% formaldehyde was used to crosslink the homogenate from the spinal cord at room temperature for 10 min, and 0.25 M glycine was added to stop reaction. The pellet was harvested and lysed in SDS lysis buffer with a protease inhibitor cocktail after centrifugation, and subjected to sonicate to shear the DNA into fragments with 200–1000 nt length. Protein G magnetic beads were added to pre-clean the fragments, and then the fragments were immunoprecipitated with 2.5 μg of rabbit anti-ETS1 antibody or normal rabbit serum overnight at 4°C. 10%–20% of immunoprecipitation sample was applied as the input positive control. The ChIP DNA fragments were purified and amplified by real-time quantitative PCR using primers in Supplemental Table S2.

### Luciferase assay

The 1174-bp fragment of *Fto* gene promoter bound by ETS1 (three motif sites) was amplified by using mouse genomic DNA (primers seen in Supplemental Table S2), and inserted into pGL6 reporter plasmid after digestion with KpnI and HindIII enzymes. The constructs were confirmed by Sanger sequencing. One day after culturing HEK293 cells in 12-well plates, each well was co-transfected with 300 ng of ETS1 overexpression plasmid, 300 ng reporter plasmid or pGL6-Basic plasmid and 10 ng of the pRL-TK (Promega) interference control with Lipofectamine 6000 (C0526, Beyotime). After 2 days’ transfection, the transfection cells were harvested and subjected to lysis in lysis buffer. After centrifugation, luciferase activity of 100 μl of supernatant was measured by using Renilla-Lumi Luciferase Reporter Gene Assay Kit (RG062S, Beyotime). The experiments were repeated three times. The relative activity of reporter was obtained through comparing the firefly activity to the renilla activity.

### mRNA stability assay

The measurement of new RNA synthesis was carried out using actinomycin-D treatment by the previous described.^21^ After intrathecal injection of si-Ythdf2 for 3 days, mice were intrathecal injected using actinomycin-D (5 μg/mL). Spinal cords were collected at 3 and 6 hours after actinomycin-D treatment. RNA was extracted for RT-qPCR to calculate the mRNA expression and mRNA decay half-life of each group.

### CRISPR vector for specific targeting of the m^6^A site in the 3’UTR of MrgprF

The dCasRx and *Fto* or *Mettl3* fusion vector in Lenti-CRISPR was constructed as described previously,^28^ with few modifications. Briefly, full-length *Fto* or *Mettl*3 was amplified from mouse cDNA and then ligated into the BamHI treated EF1a-dCasRx-2A-EGFP vector with ClonExpress II One Step Cloning kit (C112, Vazyme). Lentivirus gRNA including gRNA-490 (targeting 490 to 510 of *MrgprF* 3’UTR, first nucleotide in 3’UTR designated as 11), gRNA-611 (targeting 611 to 631), or negative control gRNA were constructed by ligating to pLH-sgRNA1, as described previously. In brief, forward and reverse oligos (Supplementary Table S2) were annealed and ligated to single BbsI-digested pLH-sgRNA1 vectors. The lentivirus package and titer identification are described in the Plasmid constructs section, above. Lentivirus-dCasRx-Fto or Lentivirus-dCasRx-Mettl3 and Lenti-gRNA were co-injected and then used in experiments after the verification of their effectiveness.

### Metabolomics analysis

To identify *Bacteroides fragilis*-driven metabolites playing active roles in itch development, we identified the metabolites respectively from plasma, spinal dorsal horn, and feces after oral gavage of *Bacteroides fragilis* in germ-free mice. As previous described,^31^ 400 μL of cold methanol/acetonitrile (1:1, v/v) was added to 50 mg blood sample to remove the protein and extract the metabolites. When extracting the spinal cords or feces metabolites, 1000 μL of cold methanol/acetonitrile/water (2:2:1, v/v) was added to  ~  80 mg sample and the extraction solvent was fully whirled. The samples were homogenized by MP homogenizer (24  ×  2, 6.0 M/S, 60 s, 2 times), ultrasonic at 4℃ (30 min/once, 2 times). Stable isotope internal standard stock solution was added to the extraction solvent for absolute quantification of metabolites. After centrifugation at 14,000 g for 20 min at 4℃, the supernatant was collected and dried in a vacuum centrifuge at 4℃. Liquid chromatography tandem mass spectrometry (LC-MS/MS) test and data analysis were performed in Shanghai Applied Protein Technology Co., LTD.

In LC-MS/MS test, the samples were redissolved in 100 μL acetonitrile/water (1:1, v/v) solvent. After centrifuging at 14,000 g at 4℃ for 15 min, the supernatant was then added into UHPLC (1290 Infinity LC, Agilent Technologies) coupled to QTRAP MS (6500 + , Sciex). The analytes were separated by HILIC (Waters UPLC BEH Amide column, 2.1 mm  ×  100 mm, 1.7 µm) and C18 (Waters UPLC BEH C18–2.1  ×  100 mm, 1.7 μm). 6500 + QTRAP (AB SCIEX) was performed in positive and negative switch mode. Polled quality control (QC) samples were set in the sample queue to assess the stability and repeatability of the system. Quantitative data processed by using MultiQuant and Analyst. Metabolites in QCs with a coefficient of variation (CV) of less than 30% were labeled as repeatable.

### Metabolites administration

A group of adult male mice after antibiotic treatment received a continuous intraperitoneal injection of Acetyl-l-carnitine (MCE 14,992-62-2) (100 mg/kg) or 1-arachidoyl-2-hydroxy-sn-glycero-3-phosphocholine (Aladdin 108,341-80-6) (50 mg/kg) for 5 days. Both drugs were dissolved using DMSO and each mouse was injected with 0.2 ml. The control group was injected with DMSO. Mice were received with normal drinking water during the intraperitoneal injection treatment.

### Experimental design and statistical analysis

Based on the literature of the field and our previous experience,^21,32^ the size of the experimental groups was determined. Each experiment presented in the study was repeated in multiple animals (three to eight mice per sample, see relevant sections). In all experiments, animals were randomly allocated to the experimental groups. To achieve specific regulation of gene expression in the spinal cord, intrathecal injection of viruses or siRNAs was employed, as previously described.^21,33^ Double-blind testing was implemented in all behavioral experiments. Furthermore, to eliminate the potential influence of locomotor impairment on itch behavior data, locomotor function was assessed as part of this study. Statistical analyses were carried out as described.^28^ All data are presented as the mean ± SEM or as heat maps. Significance testing was performed with one-way or two-way ANOVAs or with paired or unpaired Student’s t tests. *p* < 0.05 was considered statistically significant in all analyses.

## Results

### Mice lacking microbiota exhibit tolerance to itch

**Figure 1.**
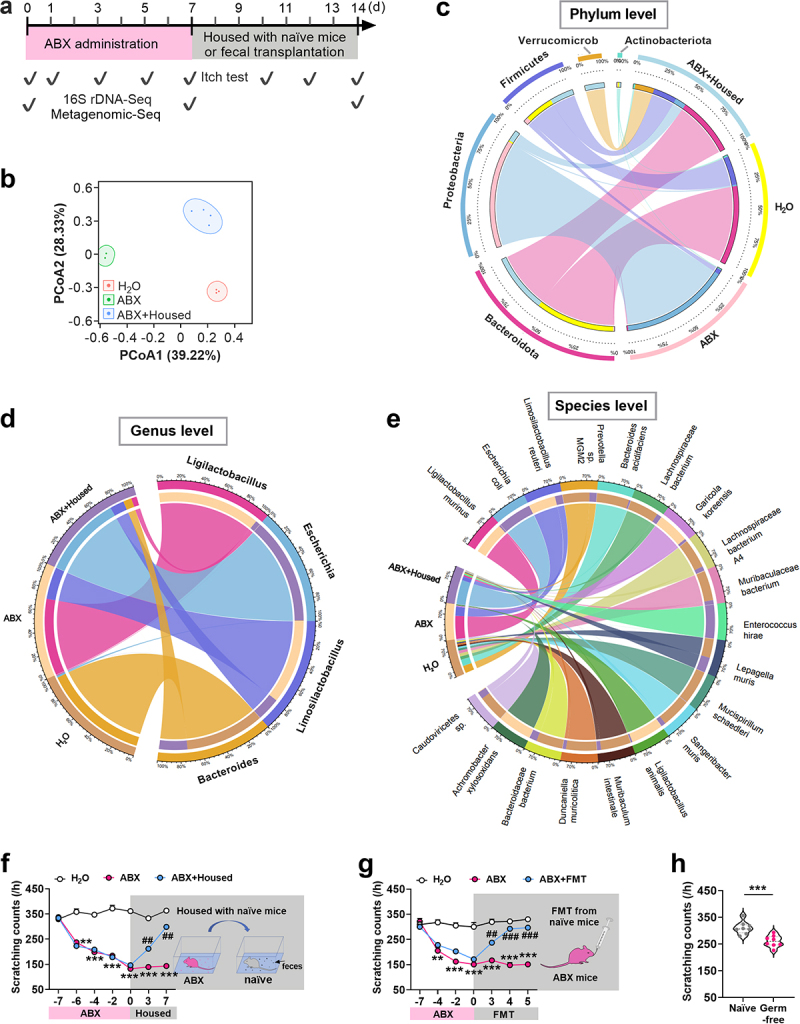
The tolerance to itch occurs after depletion of the gut microbiota. (a) time line and schedule showing treatment with antibiotics (ABX), housing conditions (Abx + housed), itch behavior test sessions, 16S rDNA gene sequencing (16S rDNA-seq), and metagenomic sequencing (Metagenomic-Seq). (b) Principal coordinates analysis (PCoA) of gut microbiota composition for three groups: naïve (H_2_O), ABX, and ABX + housed through 16S rDNA sequencing. *n* = 3 or 4 mice/group. Please note that two of the three data points from the ABX samples are too close together, resulting in overlapping markers in the plot. (c) Circos analysis displaying the corresponding abundance relationship between samples and bacterial communities at phylum level via 16S rDNA sequencing. *n* = 3 or 4 mice/group. (d, e) the circos analysis was performed on the genus (d) and species (e) of bacteria that exhibited higher abundance on day 7 after being housed in the gut of ABX-treated mice, utilizing metagenomic sequencing. *n* = 4 mice/group. f, housed with naïve mice blocks a tolerance to itch induced by reduction of gut microbiota. Scratching bouts (/h) in response to chloroquine from day 0 to day 14 after the start of ABX treatment (or water control) in naïve adult mice, and from day 7 to day 14, when mice were housed with naïve mice. **p < 0.01, ***p < 0.001 versus H_2_O control group; ^##^*P* < 0.01 versus ABX, two-way ANOVA, *n* = 8 mice/group. (g) Fecal microbiota transplantation (FMT) from naïve adult mice inhibits the ABX-caused tolerance to itch. **p < 0.01, ***p < 0.001 versus H_2_O; ^##^*P* < 0.01, ^###^*P* < 0.001 versus ABX, two-way ANOVA, *n* = 8 mice/group. Fecal microbiota of naïve mice was transplanted into the ABX mice via oral gavage starting from day 0. (h) the analysis of scratching bouts in response to chloroquine in adult male germ-free mice. ***p < 0.001 versus naïve male mice. Student t’ test. *n* = 8 mice/group.

To investigate the potential role of gut microbiota in itch, we assessed scratching responses in a chloroquine-induced chemical itch model. Gut microbiota depletion was achieved by feeding mice a combination of four different antibiotics (ABX mice) for 7 days, targeting various bacterial species. Subsequently, microbiota restoration was performed by co-housing ABX mice with normal mice (ABX + housed mice) for 7 days, allowing the ABX mice to consume feces from normal mice and thereby reestablish their gut microbiota. To further evaluate the impact of ABX treatment on gut microbiota, fecal samples were collected and analyzed for changes in bacterial composition using 16S rDNA sequencing and metagenomics sequencing (Figure 1(a)). Compared to the control group (drinking H_2_O), ABX mice exhibited a significant reduction in the richness and diversity of gut microbiota communities. This reduction was partially reversed after co-housing with normal mice. (Figure 1(b) and Supplemental Figure S1a-f), These results indicate that antibiotic administration resulted in a substantial depletion of gut microbiota, while co-housing treatment partially restored the lost microbial communities. The bacteria that exhibited significant changes in abundance were primarily concentrated in five phyla: *Bacteroidota, Proteobacteria, Firmicutes*, *Verrucomicrobiota* and *Actinobacteriota* after ABX treatment (Figure 1(c)). Metagenomic sequencing further revealed that these altered bacteria were predominantly distributed across four genera (Figure 1(d)) and nineteen species (Figure 1(e)) after ABX treatment. Notably, *Bacteroidota* was identified as the second most abundant phylum in the normal control group. It exhibited the greatest change following antibiotic treatment and recovered the fastest after co-housing (Figure 1(c)).

To measure itch, mice were intradermally (*i.d*.) injected with chloroquine in the nape of the neck and their scratching behavior was monitored for 1 h after the injection.^32^ Chloroquine-evoked scratch bouts were significantly reduced from day 1 to day 7 (day  − 6 to 0 in axis) after ABX treatment, compared with the control H_2_O group (Figure 1(f)). However, chloroquine-induced scratch bouts in ABX mice were observed to increase by the third day of co-housing with normal mice (day 3) and gradually returned to baseline levels by the seventh day (day 7) (Figure 1(f)), indicating the restoration of normal itch sensation. To exclude the possible effect of metabolite in feces on itch behavior, we isolated gut microbiota from the feces of receptor naïve mice to oral gavage the ABX-treated mice. The similar restoration of itch tolerance induced by ABX was observed during the period from day 3 to 5 after the transplantation of the isolated gut microbiota (Figure 1(g)). Given that antibiotics may have potential effect on itch behavior, adult germ-free mice were used to evaluate the change of itch behavior. The scratching frequencies to chloroquine stimulus from germ-free mice was significantly decreased compared to naïve mice (Figure 1(h)). However, we did not observe any impairments in locomotor function after the loss of gut microbiota or germ-free (Supplemental Table S1). These data indicate that depletion of the gut microbiota enhances tolerance to itch. Thus, the gut microbiota appears to play an important role in eliciting the itch sensation.

### Deletion of gut microbiota causes the decrease of FTO in the dorsal spinal horn

**Figure 2.**
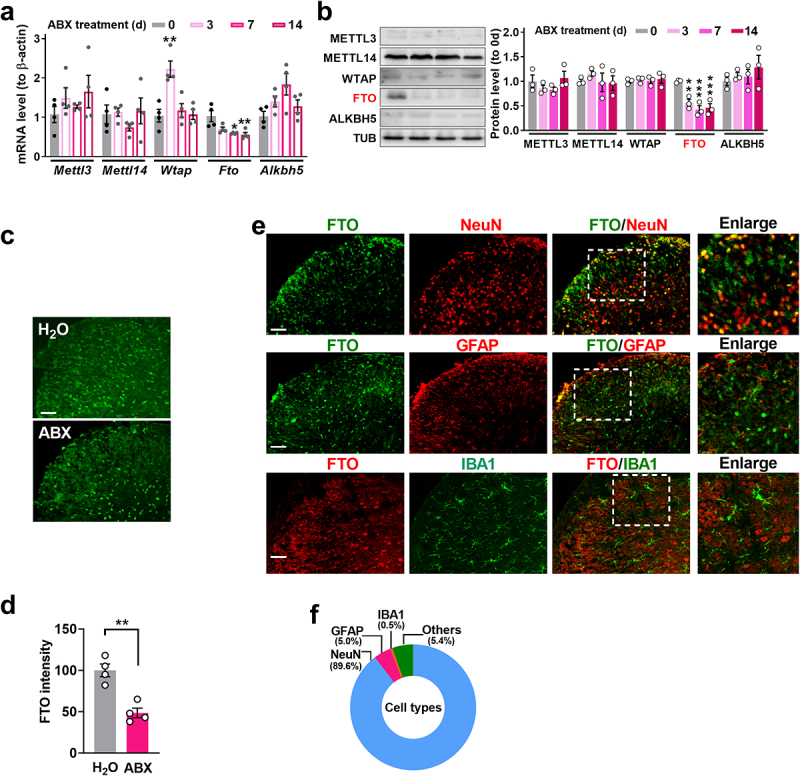
Deletion of the gut microbiota decreases the level of FTO expression in the dorsal horn. (a) the mRNA expression of m^6^A methyltransferases (*Mettl3*, *Mettl14*, and *Wtap*) and demethylases (*Fto* and *Alkbh5*) in the dorsal horn in mice lacking microbiota. *Fto*: **p < 0.0001, two-way ANOVA, post hoc Tukey’s test. *n* = 4 mice/group. (b) the level of METTL3, METTL14, and WTAP and FTO and ALKBH5 protein in the dorsal horn in mice lacking microbiota. ***p < 0.05, two-way ANOVA, post hoc Tukey’s test. *n* = 3 mice/group. (c, d) FTO expression in the dorsal horn on day 7 after ABX treatment. *n* = 4 mice (biological repeats). Scale bar, 50 μm. (e, f) FTO (green) distribution in different cellular types: neuron (red, NeuN), microglia (red, Iba1), astrocytes (red, GFAP). *n* = 4 mice (biological repeats). Scale bar, 50 μm.

RNA m^6^A modification plays an established role in CNS dysfunction, including chronic pain, inflammation, and Alzheimer’s disease. Because the spinal cord is a key signal-processing step in the neural pathway underlying itch,^32,34^ we examined the expression of m^6^A RNA methyltransferases (METTL3, METTL 14, and WTAP) and demethylases (FTO and ALKBH5) in the L3/4 dorsal horn of mice lacking the gut microbiota. We found that *Fto* mRNA expression was decreased in a time-course-dependent manner: by 30% on day 3, by 39% on day 7, and by 51% on day 14 after initiation of ABX treatment, compared to the control group (day 0) that did not undergo ABX treatment. (Figure 2(a)). However, except for an increase in *Wtap* mRNA on day 3 and a slight increase in *Alkbh5* mRNA on day 7, we did not observe any changes in *Mettl3* and *Mettl14* mRNA in the dorsal horn during the observation period after treatment (Figure 2(a)). Notably, the expression level of FTO protein was dramatically decreased in the L3/4 dorsal horn from day 3 to 14 after ABX (Figure 2(b)), but the expression levels of the other four proteins (METTL3, METTL14, WTAP, and ALKBH5) were unchanged after ABX, compared to the control mice (Figure 2(b)). Furthermore, immunofluorescent staining confirmed the ABX-induced decrease in FTO protein in the dorsal horn on day 7 after ABX treatment, compared to the control group (Figure 2(c,d)). Co-staining showed that 89.6% of FTO-positive cells were also positive for NeuN (a specific neuronal marker), and about 4.5% of were positive for GFAP (a marker of glial cells), but almost none were also positive for IBA1 (a marker for microglial) (Figure 2(e,f)), indicating that FTO is predominantly expressed in spinal neurons. the level of m^6^A modification on RNA exhibited a time-dependent increase. Given that FTO functions as an m^6^A RNA demethylase, we investigated changes in global RNA m^6^A levels in the spinal cord following gut microbiota depletion. We observed that, in contrast to the expression of FTO, global RNA m^6^A levels were significantly elevated on days 3, 7, and 14 post-ABX treatment in the L3/4 dorsal horn, compared to the day 0 control group. (Supplemental Figure 2(a)). These results strongly suggest that FTO may be a critical regulator in the tolerance of itch induced by gut microbiota depletion.

### ETS1 inhibits spinal Fto transcription activity after deleting gut microbiota

**Figure 3.**
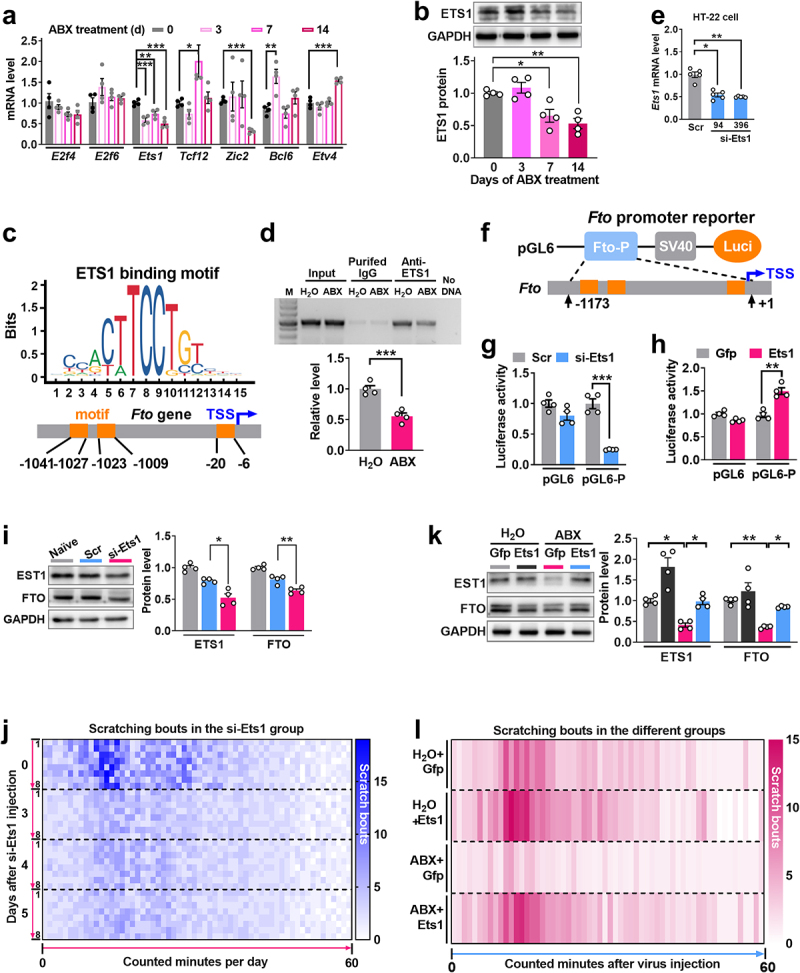
ETS1 downregulation inactivates FTO expression in the dorsal horn following depletion of gut microbiota. (a) the expression level of seven consensus transcription factors–*E2f4*, *E2f6*, *Ets1*, *Tcf12*, *Zic2*, *Bcl6*, and *Etv4*—in the dorsal horn on days 3, 7, and 14 after the start of ABX treatment. **p < 0.05, ***p < 0.01 versus day 0, two-way ANOVA, post hoc Tukey’s test. *n* = 4 mice/group. (b) ETS1 protein level on days 3, 7, and 14 after the start of ABX treatment in the dorsal horn. ^##^*P* < 0.05, **p < 0.01 versus day 0, one-way ANOVA test post hoc Tukey’s test. *n* = 4 mice/group. (c) two ETS1 binding motif regions located, respectively, at  −  1041 to  −  1009 and  −  20 to  −  6 of the mouse *F to* promoter (transcription start site designated as  +  1). (d) *Fto* promoter fragment immunoprecipitated by rabbit anti-ETS1 antibody in the ipsilateral dorsal horn on day 7 after the start of ABX treatment. Input: total purified fragments. IgG: purified rabbit IgG. M: DNA ladder marker. ***p < 0.05 versus the H_2_O group; two-tailed unpaired Student’s t test. *n* = 4 mice/group. e, the expression of *Ets1* mRNA in HT-22 cells at 48 h after injection of the *Ets1* siRNA (si-Ets1) si-94 and si-396. ^##^*P* < 0.05 versus Scr, one-way ANOVA test, post hoc Tukey’s test. *n* = 5. f, luciferase reporter construction. pGL6: empty vector. Fto-P, *fto* promoter containing  −  1173 to  +  1 (the first nucleotide of transcription start site is designed as  +  1). g, h *Fto* promoter activity at 36 h in HEK-293T cells transfected with *Ets1* siRNA (g) or at 48 h with *Ets1* full-length expression plasmid (h). Scr: Scrambled siRNA treatment. si-Ets1, si-Ets1–94 treatment. pGL6: empty vector control. pGL6-P: pGL6 reporter with *fto* promoter. Ets1: Lentivirus expressing full-length *Ets1*. Gfp: lentivirus expressing *Gfp* control. ^###^*P* < 0.05 versus Scr (g) or Lenti-Gfp (h); two-tailed unpaired Student’s t test. *n* = 4. i, the expression of ETS1 and FTO protein in the dorsal horn on day 3 after intrathecal injection of *Ets1* siRNA or its control scrambled siRNA. ***p < 0.001, one-way ANOVA test, post hoc Tukey’s test. *n* = 4 mice/group. (j) Heat-map analysis of scratching bouts in response to chloroquine in naïve mice after intrathecal injection of *Ets1* siRNA. The left 0, 3, 4 and 5 refer to days after siRNA injection. The right from 1 to 8, the individual 8 mice. k, Level of ETS1 and FTO protein in the dorsal horn on day 5 after Lenti-Ets1 or Lenti-Gfp injection in ABX with 7 days treatment. ***p < 0.05, *p < 0.01 versus corresponding groups, one-way ANOVA test, post hoc Tukey’s test. *n* = 4 mice/group. (l) Heat-map analysis of scratching bouts in response to chloroquine in ABX mice on day 5 after intrathecal injection of Lenti-Ets1 or Lenti-Gfp. *n* = 6 mice/group.

Next, we investigated the mechanism underlying the inhibition of spinal FTO expression following gut microbiota depletion. The online TFSEARCH program was used to identify potential transcription factors regulating *Fto* transcription. Seven consensus transcription factors—*E2f4*, *E2f6*, *Ets1*, *Tcf12*, *Zic2*, *Bcl6*, and *Etv4*—had binding motifs with a relatively high score in the *Fto* promoter. Of these, two (*Ets1* and *Zic2*) were significantly downregulated on day 14 after ABX treatment in the dorsal horn, relative to the day 0 control group and only *Ets1* showed time-course-dependent changes consistent with the changes in *Fto*. Three genes (*Tcf12* on day 7, *Bcl6* (transiently) on day 3 and *Etv4* on day 14) were significantly upregulated, and two genes (*E2f4* and *E2f6*) showed no change in expression after ABX treatment, relative to the control group (Figure 3(a)). Based on these results, *Ets1* was selected as the candidate target in the following experiments. Dorsal horn ETS1 protein expression was significantly reduced on day 7 and day 14 after the start of ABX administration, but not on day 3, compared to the control group (Figure 3(b)). To determine whether *Ets1* regulates *Fto* expression, we examined their binding *in vitro*. A chromatin immunoprecipitation (ChIP) assay showed that the binding activity of ETS1 to the *Fto* promoter was reduced on day 5 after ABX, as demonstrated by a 1.5-fold decrease in the band density in spinal cord from ABX-treated mice compared with that from H_2_O-treated mice (Figure 3(c,d)). The reduced binding activity may be attributed to the decrease in ETS1 expression levels in the dorsal horn after ABX (Figure 3(d)).

To evaluate whether *Ets1* regulates *Fto* expression, we synthesized *Ets1* siRNAs and constructed a Lenti-*Ets1* vector containing the full length *Ets1*. Because the *Ets1* gene is highly conserved between human and mouse, two synthesized *Ets1* siRNAs were targeted to two mouse *Ets1* mRNA regions (1^st^: 94  ~  112; and 2^nd^: 396  ~  414, the first nucleotide of CDS designated as  +  1) and human *Ets1* mRNA regions (CDS: 226  ~  244; 613  ~  631). We found that *Ets1*-siRNA-94 and *Ets1*-siRNA-396 knocked down *Ets1* mRNA expression by 48% and 53%, respectively, relative to the Scrambled (Scr) group, 36 h after transfection in HT-22 cells (Figure 3(e)). To further demonstrate the regulatory role of ETS1 in *Fto* expression, we constructed a luciferase reporter pGL6-*Fto* with a 1174 bp-length Fto promoter including three predicted binding motif sites (Figure 3(f)). The luciferase assay revealed that co-transfection of *Ets1* siRNA, but not scrambled siRNA, with pGL6-*Fto* significantly reduced the activity of the luciferase reporter 36 h after transfection in HEK293 cells (Figure 3(g)). Conversely, co-transfection of Lenti-*Ets1*, but not the control Lenti-*Gfp* (Gfp), enhanced the luciferase activity of pGL6-*Fto* 36 h after transfection in HEK293 cells (Figure 3(h)). As expected, intrathecal injection of *Ets1* siRNA decreased the level of both ETS1 and FTO protein expression on day 3 after injection in naïve mice, compared with Scr control (Figure 3(i)), resulting in the decreased bouts of scratching to chloroquine, compared to the day 0 control group (Figure 3(j)). We did not observe any impairments in locomotor function (Supplemental Table S1). In contrast, when compared to Lenti-Gfp, intrathecal injection of Lenti-*Ets1* prevented the ABX-induced decrease in both ETS1 and FTO protein in the dorsal horn, measured on day 5 after injection and day 3 after ABX treatment (Figure 3(k)); behaviorally, ETS1 upregulation prevented the decrease in scratching bouts in response to chloroquine in ABX mice (Figure 3(l)). Again, none of these treatments affected locomotor function (Supplemental Table S1). Our findings strongly support the hypothesis that *Ets1* participates in loss of *Fto* induced by gut microbiota depletion in the dorsal horn.

### Restored FTO expression in dorsal horn weakens microbiota deletion-induced itch tolerance

**Figure 4.**
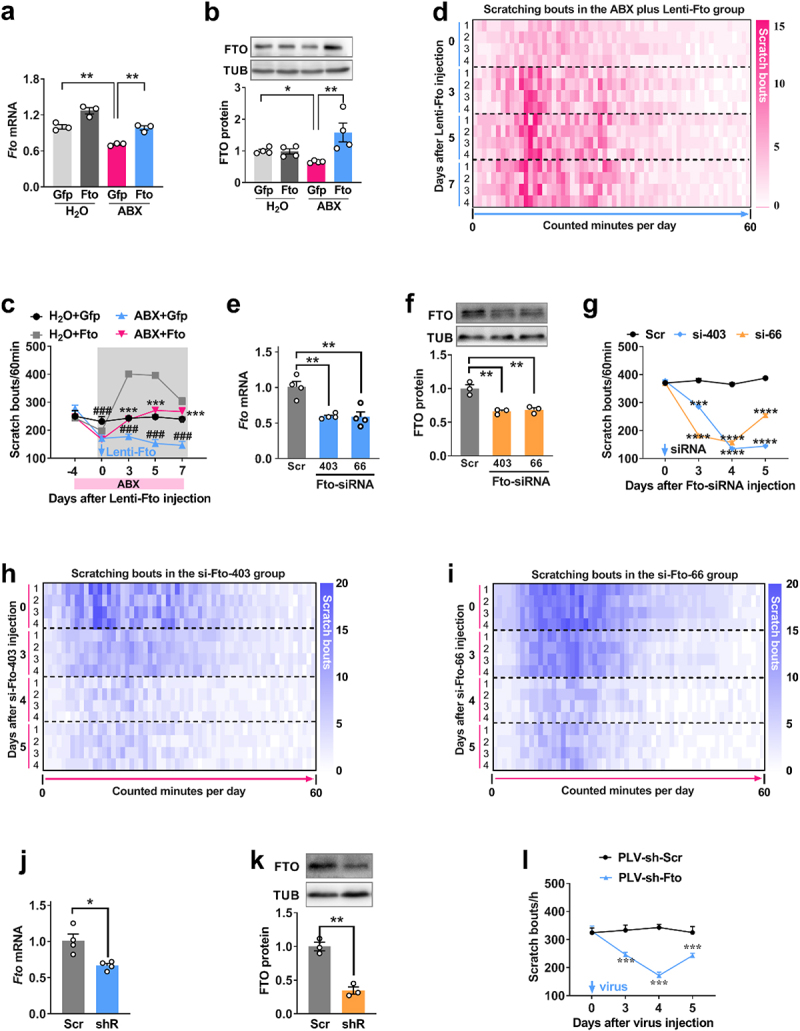
Rescuing the decrease in dorsal horn FTO alleviates itch tolerance induced by depletion of gut microbiota. (a, b) the level of *Fto* mRNA (a) and protein (b) in the dorsal horn on day 5 after intrathecal injection of Lenti-*Fto* (Fto) or Lenti-*Gfp* (Gfp) in mice treated with ABX for 7 days. **p < 0.05, ***p < 0.01, ^##^*P* < 0.001 versus corresponding groups, one-way ANOVA, post hoc Tukey’s test. *n* = 4 mice/group. c, time course of the effect of injection of Lenti-*Fto* (Fto) or Lenti-*Gfp* (Gfp) on scratching bouts to chloroquine in mice treated with ABX. **p < 0.01 versus H_2_O + Gfp; ***p < 0.01, ^##^*P* < 0.001 versus ABX + Gfp at the corresponding time points; two-way ANOVA, post hoc Tukey’s tests. *n* = 8 mice/group. Red bar, ABX or H_2_O administration. Blue arrow, lenti-*fto* or Lenti-*Gfp* injection. d, Heat-map analysis of scratching bouts to chloroquine on day 5 after intrathecal injection of Lenti-*Fto* (Fto) in mice treated with ABX. The left 0, 3, 4 and 5 refer to days after Lenti-Fto injection. The right 1, 2, 3 and 4, the repeated 4 times. (e, f) The level of *fto* mRNA (e) and protein (f) in mice after intrathecal injection of *Fto* siRNA  −  403 or  −  66. ^###^*P* < 0.001 versus scr, one-way ANOVA, post hoc Tukey’s tests. *n* = 3–4 mice/group. g, Time course of the effect of injection of *fto*-siRNA (siR) or scrambled siRNA (scr) on scratching bouts to chloroquine in naïve mice. ***p < 0.05, ***p < 0.01 versus the scr group at the corresponding time points. Two-way ANOVA, post hoc Tukey’s tests. *n* = 7 mice/group, blue arrow, *Fto*-siRNA or Scr injection. (h, i) Scratching bouts to chloroquine in naïve mice on days 3, 4, and 5 after intrathecal injection of *Fto* siRNA-403 (h) or  −  66 (i). The left 0, 3, 4 and 5 refer to days after siRNA injection. The right 1, 2, 3 and 4, the individual 4 mice. j, k, Dorsal horn *Fto* mRNA (j) and protein (k) in mice after intrathecal injection of Lenti-*Fto*-shRNA (shRNA) or Lenti-Scr-shRNA (Scr). *p < 0.01, *p < 0.001 versus Scr, two-tailed unpaired Student’s t test, *n* = 3–4 mice/group. l, Time course of scratching bouts to chloroquine in naïve mice after intrathecal injection of *Fto*-shRNA (shRNA) or Lenti-Scr-shRNA (Scr). **p < 0.05, *p < 0.01 versus Scr, two-way ANOVA, post hoc Tukey’s tests. *n* = 7 mice/group.

To evaluate the contribution of FTO to itch, we constructed a lentivirus vector to overexpress full-length *Fto* in the dorsal horn of mice lacking gut microbiota and then subjected the mice to the previously described itch behavioral test.^35^ Intrathecal injection of Lenti-*Fto* in ABX mice only rescued the decrease in dorsal horn *Fto* mRNA (Figure 4(a)) and protein (Figure 4(b)), but also prevented the elevation in global RNA m^6^A level (Supplemental Figure S2b), compared to the Lenti-*Gfp* control, as measured on day 5 after the Lenti-Fto injection (day 9 after the start of ABX treatment). Behaviorally, the upregulation of FTO in ABX mice as well as abolished the increased itch tolerance, as shown by increased chloroquine-induced scratching from day 3 after injection (which occurred after 3 days of ABX pre-treatment) (Figure 4(c,d)). This reversal lasted at least 7 days after lentivirus injection.

Conversely, to investigate whether dorsal horn *Fto* knockdown leads to itch tolerance in naïve mice, we employed two knockdown tools: exogenous siRNA and endogenous shRNA. Two *Fto* siRNAs (targeting mouse CDS 1^st^: 66  ~  84 and 2^nd^: 403  ~  422, respectively; the first nucleotide of CDS designated as  +  1) were designed and injected intrathecal in naïve mice. *Fto*-siRNA-66 and *Fto*-siRNA-403 reduced *Fto* mRNA by 39% and 40.5%, respectively, in the dorsal horn on day 3 after injection, compared to the Scr group (Figure 4(e)). Consistent with this, siRNA-66 and siRNA-403 injection also knocked down dorsal horn *Fto* protein by 27% and 28.5%, respectively, on day 3 post-injection, relative to the Scr control (Figure 4(f)). Notably, *Fto* downregulation led to a robust increase in spinal m^6^A—130% after siRNA-66 injection and 141% after siRNA-403 injection (Supplemental Figure 2(c)). Moreover, we further observed that downregulation of *Fto* from both siRNAs caused significant decreases in chloroquine-induced scratching from day 3 after injection, and this reduction of scratching lasted until at least day 5 after injection (Figure 4(g–i)). To further confirm the effect of *Fto* knockdown on global RNA m^6^A content, we constructed Lenti-*Fto*-shRNA. Similar to the results with siRNA, intrathecal injection of Lenti-*Fto*-shRNA in naïve mice significantly decreased *Fto* mRNA (Figure 4(j)) and protein (Figure 4(k)) expression and increased the spinal m^6^A level (Supplemental Figure S2d), in the dorsal horn on day 5 after injection, compared to the scrambled shRNA control. The number of scratch bouts also diminished starting from day 4 after shRNA injection, a degree of itch tolerance was also observed on day 5 after intrathecal injection of Lenti-*Fto*-shRNA (Figure 4(l)). Our results suggest that spinal *Fto* plays an essential role in the initiation of itch in the RNA m^6^A-dependent manner.

### FTO modulates MrgprF through an RNA m^6^A-dependent mechanism

**Figure 5.**
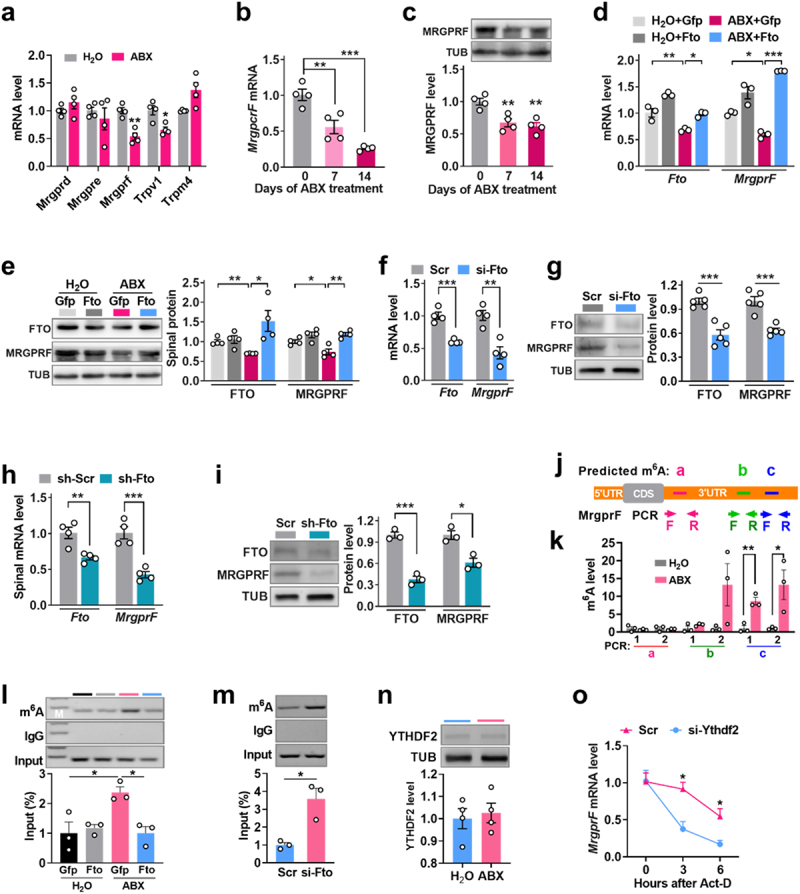
ABX-induced downregulation of FTO results in m^6^A-dependent downregulation of *MrgprF*. (a) the levels of *MrgprB*, *MrgprE*, *MrgprF*, and *Tripv1* mRNA in the dorsal horn on day 7 of ABX treatment. **p < 0.01 versus Scr, two-tailed unpaired Student’s t test. *n* = 4 mice/group. b, c Time-course analysis of *MrgprF* mRNA (b) and protein (c) expression in the dorsal horn after ABX treatment. ***p < 0.05, ^##^*P* < 0.01, **p < 0.001versus day 0, one-way ANOVA, post hoc Tukey’s tests. *n* = 4 mice/group. d, e, the levels of *Fto* and *MrgprF* mRNA (d) and protein (e) in the dorsal horn on day 5 after intrathecal injection of Lenti-*Fto* (Fto) or Lenti-*Gfp* (Gfp) in mice treated with ABX for 7 days. ***p < 0.05, ^##^*P* < 0.01, ^###^*P* < 0.001 versus the corresponding groups, one-way ANOVA, post hoc Tukey’s tests. *n* = 3–4 mice/group. (f, g) the level of dorsal horn *fto* and *MrgprF* mRNA (f) and protein (g) on day 2 after intrathecal injection of *Fto*-siRNA (si-*Fto*) or scrambled siRNA (Scr) in naïve mice. ***p < 0.01, ***p < 0.001 versus Scr groups, two-tailed unpaired student’s t test. *n* = 4–5 mice/group. (h, i) the level of *fto* and *MrgprF* mRNA (h) and protein (i) in the dorsal horn on day 5 after intrathecal injection of Lenti-*Fto*-shRNA (sh-*Fto*) or scrambled siRNA (sh-Scr) in naïve mice. *p < 0.01, *p < 0.001 versus sh-Scr groups, two-tailed unpaired Student’s t test. *n* = 3–4 mice/group. j, the predicted location of the m^6^A motif (GGACR. R represents A, C, or U) in the 3’UTR of *MrgprF* mRNA. The forward and reverse arrows represent paired RT-PCR primers. a-c, the three m^6^A motif regions in the *MrgprF* 3’UTR. F and R, the designed PCR primer pairs to amplify the specific m^6^A motifs. k, Detection of the three corresponding m^6^A motifs on day 7 after ABX treatment via RNA immunoprecipitation (RIP)-PCR using the three PCR primer pairs. **p < 0.01 versus Scr, two-tailed unpaired Student’s t test. *n* = 3 mice/group. l, RIP-PCR with anti-m^6^A revealed the m^6^A level at the *MrgprF* 3’UTR (c motif site in Figure 5(j)) on day 5 after intrathecal injection of Lenti-*Fto* (Fto) or Lenti-G*fp* (Gfp) in mice treated with ABX for 7 days. *p < 0.001 versus corresponding groups, one-way ANOVA, post hoc Tukey’s tests. *n* = 3 mice/group. m, *MrgprF* m^6^A level on day 2 after injection of *Fto*-siRNA or Scr control in naive mice. **p < 0.01 versus Scr, two-tailed unpaired student’s t test. *n* = 3 mice/group. n, the level of YTHDF2 protein in the dorsal horn on day 7 after the start of ABX treatment. *n* = 4 mice/group. o, *MrgprF* mRNA stability in naïve mice after Ythdf2-siRNA or Scr for 2 consecutive days, and after one-time intrathecal injection of actinomycin D (act-D, 10 mg). *p < 0.05 versus Scr, two-way ANOVA, post hoc Tukey’s tests. *n* = 3 mice/group.

Next, we explored how *Fto* participates in the pathways underlying itch dysfunction after depletion of gut microbiota. Given that G protein-coupled receptors and transient receptor potential (TRP) cation channels are associated with the sensation of itch,^36,37^ and *Mrgprd*, *Mrgpre*, *Mrgprf*, *Trpv1*, and *Trpm4* mRNAs are methylated in adult mouse dorsal root ganglion, these seemed likely mechanistic candidates. To determine their possible association with gut microbiota-induced itch, we first examined their expression in the dorsal horn following gut microbiota depletion induced by ABX treatment. Depletion of gut microbiota suppressed *MrgprF* (by 46%) and *Trpv1* (by 34%) expression in the dorsal horn on day 7 after ABX treatment, but did not affect the expression of the other three genes (Figure 5(a)). The depletion of gut microbiota suppressed *MrgprF* more significantly, so no further research on *Trpv1* was conducted in the subsequent experiments. Furthermore, the expression levels of *MrgprF* mRNA (Figure 5(b)) and protein (Figure 5(c)) were observed to decrease both on day 3 and day 14 following ABX treatment. Furthermore, the ABX-induced decreases in *MrgprF* mRNA (Figure 5(d)) and protein (Figure 5(e)) were reversed by intrathecal injection of Lenti-*Fto* (on day 5 after injection), relative to Lenti-Gfp injection. Conversely, intrathecal injection of *Fto*-siRNA significantly diminished the expression of *MrgprF* mRNA (Figure 5(f)) and protein (Figure 5(g)), relative to Scr injection. Similarly, intrathecal injection of Lenti-*Fto*-shRNA also decreased *MrgprF* mRNA (Figure 5(h)) and protein (Figure 5(i)) expression, compared to the scrambled shRNA. Thus, *MrgprF* was selected as the candidate downstream target of *Fto* in the following experiments.

Next, we sought to determine whether *MrgprF* mRNA undergoes m^6^A methylation and, if so, which specific m^6^A site is linked to its MRGPRF expression. As FTO functions to “erase” RNA m^6^A through recognizing and binding to the “GGACA/T/C” motif of target mRNA 3’UTR.^38^ We conducted bioinformatics analysis on the m^6^A motifs contained within *MrgprF* mRNA 3’UTR, we saw three regions that may contain the m^6^A motif (Figure 5(j)). This is consistent with the recently reported m^6^A site in *MrgprF* uncovered using m^6^A-SMART-Seq.^29^ We designed six pairs of primers to cover all of the predicted motif regions in the *MrgprF* 3’UTR [PCR; two pairs of primers for the a (163 to 167) motif, two for the b (534 to 538) motif, and two for the c (685 to 689) motif; the first nucleotide of the 3’UTR was designated as  +  1)] (Figure 5(j)). An RNA immunoprecipitation (RIP)-PCR assay revealed that two fragments containing the b and c motifs (but not the fragments containing a motif) were amplified both from the control and treatment complex immunoprecipitated with the m^6^A antibody (Figure 5(k)), however, only the m^6^A level in the c motif was increased by 80% after ABX treatment (Figure 5(k)), and this increase was reversed by FTO upregulation through intrathecal injection of Lenti-Fto, compared to Lenti-Gfp injection (Figure 5(l)). Contrarily, FTO downregulation via injection of *Fto*-siRNA led to increased m^6^A levels in the dorsal horn of naïve mice on day 2 after injection (Figure 5(m)). Additionally, considering YTHDF2 contributes to mRNA degeneration via binding to m^6^A sites in target mRNA, we further examine whether YTHDF2 participates in regulation of *MrgprF* translation efficiency in m^6^A-dependent manner. We found that ABX-caused gut microbiota depletion did not change the level of YTHDF2 in dorsal spinal horn at day 7 after administration (Figure 5(n)). To further verify the mechanism of YTHDF2 regulation of MRGPRF by decaying *MrgprF* mRNA, we tested the effect of *Ythdf2* knockdown with siRNA on *MrgprF* mRNA lifetime. Measuring the decay of *MrgprF* mRNA after blocking new mRNA synthesis with actinomycin-D showed that silencing YTHDF2 enhanced the level of total *MrgprF* mRNA (Figure 5(o)), suggesting that YTHDF2 destabilized *MrgprF* mRNA. Collectively, these data suggested that FTO participates in the regulation of m^6^A modification of *MrgprF* mRNA in YTHDF2-dependent manner.

**Figure 6.**
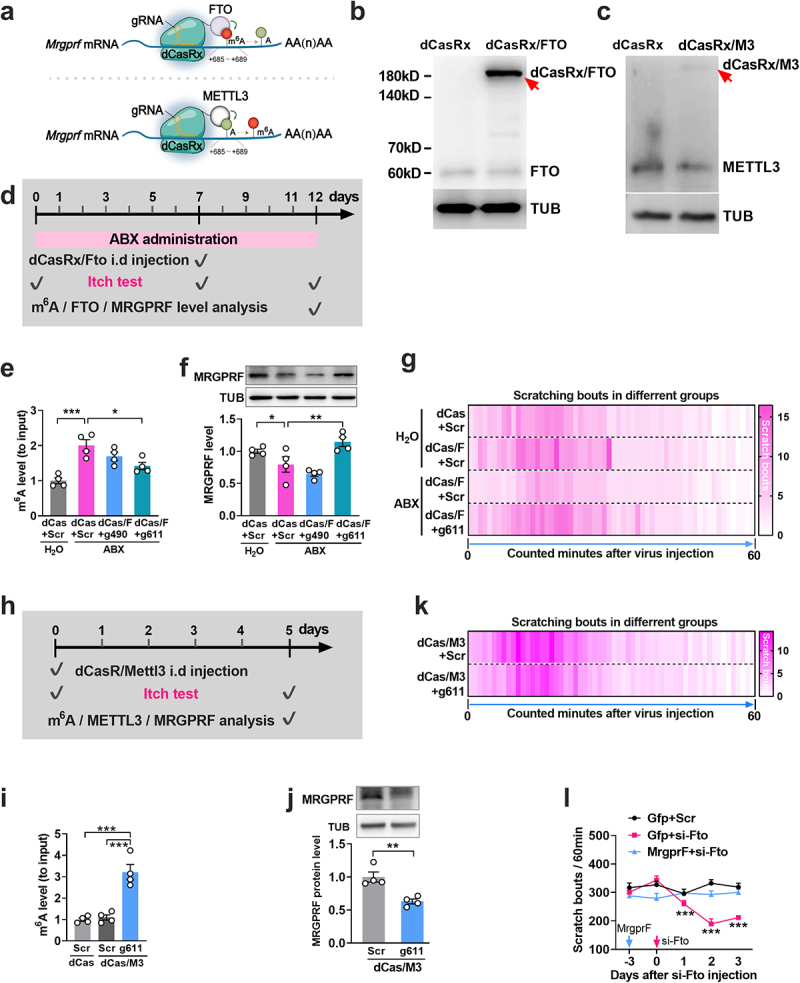
Specific regulation of m^6^A in *MrgprF* 3’UTR alters the expression of MRGPRF. (a) CRISPR-dCasrx “erasing” (upper) or “writing” (lower) m^6^A in the given 3’UTR of *MrgprF* mRNA. gRNA, small guide RNA. 685 to 689 represents the locations of the c m^6^A motif site in the *MrgprF* 3’UTR. The first nucleotide of the 3’UTR is designated as  +  1. (b, c) Identification of dCasrx-FTO (b) or dCasrx-METTL3 (c) fusion protein expression on day 5 after intrathecal injection of CRISPR-dCasrx-Fto (dCasrx/FTO) or CRISPR-dCasrx-Mettl3 (dCasrx/M3). (d) Time line and schedule showing treatment with ABX, CRISPR-dCasrx/fto injection, itch behavior test, and *MrgprF* m^6^A and protein level analysis. e, f, Analysis of m^6^A levels in *MrgprF* 3’UTR (e) and MRGPRF and FTO protein expression (f) on day 5 after co-injection of CRISPR-dCasrx-fto and gRNA-490 or gRNA-611 in mice pretreated with ABX for 7 days. **p < 0.05, ***p < 0.01 versus corresponding groups, one-way ANOVA, post hoc Tukey’s tests. *n* = 4 mice/group. gRNA-490 (490 to 510, first nucleotide in the 3’UTR is designated as  +  1) and gRNA-611 (611 to 631) were designed to bind close to the c site in the *MrgprF* 3’UTR. g, Heat-map analysis of scratch to chloroquine on day 5 after co-injection of CRISPR-dCasrx-Fto (dCas/F) and gRNA-611 or scrambled gRNA (Scr) in ABX mice. *n* = 4 mice/group. (h) Time line and schedule showing CRISPR-dCasrx/mettl3 injection, itch behavior test, and *MrgprF* m^6^A and protein level analysis. (i, j) the level of *MrgprF* m^6^A (i) and MRGPRF and METTL3 protein expression (j) on day 5 after co-injection of CRISPR-dCasrx-Mettl3 and gRNA-611 in naïve mice. ^##^*P* < 0.05, **p < 0.01 versus corresponding groups, one-way ANOVA, post hoc Tukey’s tests(i). ***p < 0.05, ^##^*P* < 0.01 versus scrambled gRNA (Scr) group, two-tailed unpaired Student’s t test (j). *n* = 4 mice/group. (k) Heat map analysis of scratch to chloroquine on day 5 after co-injection of CRISPR-dCasrx-Mettl3 (dCasR/m3) and gRNA-611 scrambled gRNA (Scr) in naïve mice. *n* = 4 mice/group. (l) Time course of the effect of upregulating MrgprF with Lenti-MrgprF on the itch tolerance induced by Fto knockdown by siRNA. ^###^*P* < 0.05, ***p < 0.001 versus the Scr + Lenti-Gfp group; ***p < 0.05 versus Fto-siRNA + Lenti-Gfp. Two-way ANOVA, post hoc Tukey’s test. *n* = 8 mice/group.

Next, we used the CRISPR gene-editing system^39^ to further confirm the specific regulatory role of FTO in *MrgprF* mRNA m^6^A level. FTO or METTL3, a RNA m^6^A methyltransferase, were respectively fused with inactivated CasRx protein (dCasRx/FTO or dCasRx/METTL3 fusion protein) to specifically “erase” or“write” m^6^A to the c motif via guide RNA (gRNA; Figure 6(a)). The dCasRx/FTO or dCasRx/METTL3 fusion protein was detectable on day 5 after injection of lentivirus CRISPR-dCasRx-Fto or CRISPR-dCasRx-Mettl3 in naïve mice (Figure 6(b,c)), confirming the successful construction of the dCasRx/FTO or dCasRx/METTL3 fusion protein. Two gRNAs [gRNA-490 (490 to 510, first nucleotide in the 3’UTR designated as  +  1) and gRNA-611 (611 to 631)] located near the C motif were designed as described previously.^28^ The ABX treatment-induced increase in *MrgprF* m^6^A levels in the dorsal horn was reduced by 16% and 29%, respectively, compared to the Scr group on day 5 after co-microinjection of gRNA-490 or gRNA-611 with CRISPR-dCasRx-Fto (Figure 6(d,e)). This demonstrates that the m^6^A modification at the C-position motif within *MrgprF* mRNA can be site-specifically regulated by FTO or METTL3. Furthermore, co-microinjection of CRISPR-dCasRx-Fto with gRNA-611, but not gRNA-490, reversed the reduction in MRGPRF expression in the dorsal horn of ABX mice on day 5 post-injection, compared to the Scr gRNA (Figure 6(f)), and abolished the increased itch tolerance in ABX mice (Figure 6(g)) on day 5 post-injection. No locomotor impairments were observed in mice subjected to any of these treatments (Supplemental Table S1). As predicted, intrathecal co-injection of CRISPR-dCasRx-METTL3 and gRNA-611 increased *MrgprF* m^6^A levels (Figure 6(h,i)), but decreased the level of MRGPRF protein (Figure 6(j)) in dorsal horn on day 5 after injection in naïve mice, and led to the itch tolerance (Figure 6(k)). Our data strongly support the hypothesis that gut microbiota depletion-induced downregulation of *MrgprF* (a key initiator of the itch sensation) is due, at least in part, to decreased m^6^A in the spinal neurons of ABX mice, and that the level of m^6^A is controlled by FTO.

### MrgprF mediates Fto-regulated itch dysfunction

Next, we sought to investigate whether *Fto* behaviorally modulates gut microbiota depletion-induced itch tolerance through *MrgprF*. First, we assessed the role of *MrgprF* in itch behavior. We observed that *MrgprF* overexpression with Lenti-MrgprF rescued the dorsal horn *MrgprF* reduction induced by gut microbiota deletion (Supplemental Figure S3a, b) on day 5 after intrathecal injection of Lenti-*MrgprF* in ABX mice, relative to Lenti-Gfp injection, and also reversed the decrease in scratching bouts from days 2 to 5 after injection (Supplemental Figure S3c, d). In contrast, both *MrgprF* siRNA-524 and  − 857 produced significant knock-down of *MrgprF* mRNA and protein (Supplemental Figure S3e, f) on day 3 after intrathecal injection in naïve mice. Behaviorally, these siRNAs also significantly diminished scratching bouts from day 3 to day 5 after injection (Supplemental Figure S3g). Our observations suggest that *MrgprF* is involved in gut microbiota-induced itch behavior. Our observations suggest that *MrgprF* is involved in gut microbiota-induced itch behavior. To examine whether *Fto*-regulated dysfunction of itch is mediated by *MrgprF*, we measured itch behavior after upregulation of *MrgprF* in naïve mice. Remarkably, compared with the Lenti-*Gfp* control, intrathecal injection of Lenti-*MrgprF* blocked the itch tolerance caused by intrathecal pre-injection of *Fto*-siRNA (Figure 6(l)). We did not observe any locomotor changes with injection of either Lenti-*MrgprF* or *Fto*-siRNA (Supplemental Table S1). Taken together, *Fto* regulates gut microbiota depletion-induced itch tolerance through *MrgprF*.

### Treatment with *Bacteroides fragilis* restored the itch behavior lost in ABX mice

**Figure 7.**
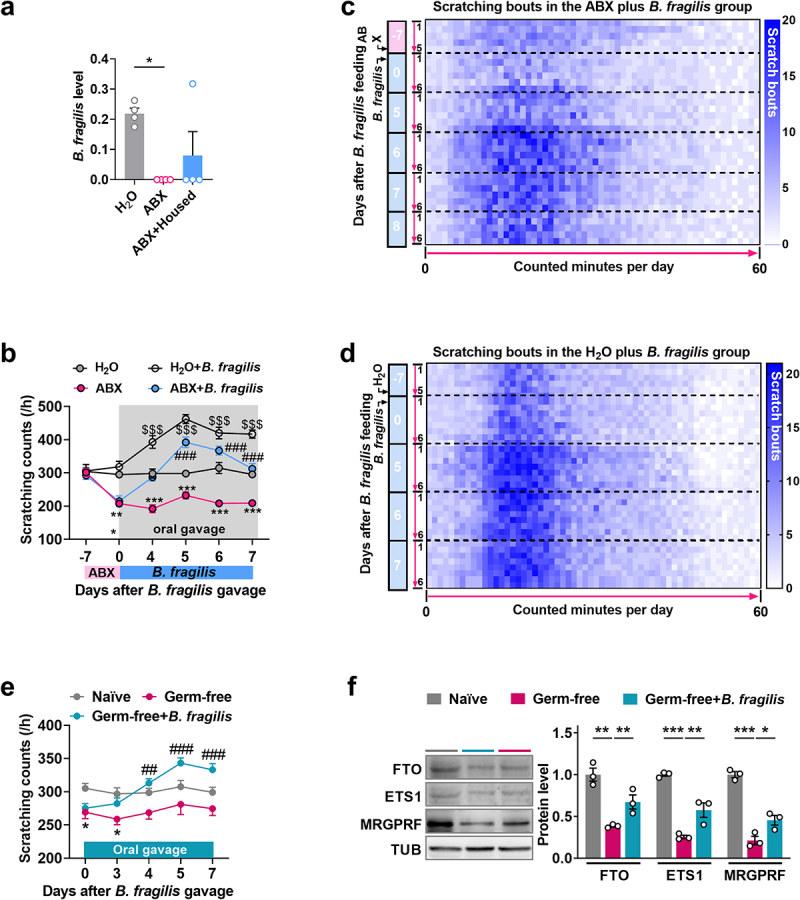
*Bacteroides fragilis* participates in modulation of itch. (a) the relative level of *B. fragilis* abundance in the gut of mice after ABX treatment and co-housing with normal mice. The data is sourced from metagenomic sequencing. **p < 0.05 versus the H_2_O group. One-way ANOVA, post hoc Tukey’s test. n = 4 mice/group. (b) Time-course analysis of the effect of oral treatment with human *B. fragilis* on itch behavior in ABX mice. ***p < 0.01, ^##^*P* < 0.001 versus the H_2_O group; **p < 0.01, ***p < 0.001 versus the ABX group; ^$$^*p* <0.01, ^$$$^*p* <0.001 versus the H_2_O group. Two-way ANOVA, post hoc Tukey’s test. n = 8 mice/group. (c) Heat-map analysis of itch behavior on day 5 after *B. fragilis* treatment in ABX mice. n = 6 mice/group. (d) Heat-map analysis of the effect on basal itch behavior on day 5 after *B. fragilis* treatment in naïve mice. n = 6 mice/group. (e) the effect of *B. fragilis* transplantation on scratching bouts to chloroquine in germ-free mice. ^##^*P* < 0.05 versus the naïve group; ^###^*P* < 0.01, ***p < 0.001 versus the germ-free group; Two-way ANOVA, post hoc Tukey’s tests. n = 8 mice/group. (f) the levels of FTO, EST1, and MRGPRF expression levels in the dorsal horn on day 7 after oral gavage of *B. fragilis* in germ-free mice. ***p < 0.05, *p < 0.01, *p < 0.001 versus the corresponding groups; One-way ANOVA, post hoc Tukey’s test. n = 3 mice/group.

Among hundreds of microbial species residing in the human gut, *Bacteroides fragilis* (*B. fragilis*) is a prominent species of the genus *Bacteroides* within the Gram-negative *Bacteroidota* phylum. Oral treatment with *B. fragilis* alleviates intestinal inflammation and ameliorates abnormal communicative, stereotyped, sensorimotor, and anxiety-like behaviors, as well as autoimmune encephalomyelitis.^8,40^ Given that *Bacteroides* is the genus with the highest total relative abundance of *Bacteroidota* in normal mice (Figure 1(d)), it is noteworthy that *B. fragilis* did not rank at the top in terms of total relative abundance in the species-level analysis based on metagenomic sequencing. However, the relative abundance of *B. fragilis* significantly decreased on day 7 following ABX administration compared to the H_2_O control. This reduction was notably reversed after ABX mice were co-housed with normal mice, although the change did not reach statistical significance (Figure 7(a)). Thus, to test whether restoring the gut microbiota could ameliorate ABX-associated itch abnormalities, we treated ABX mice or germ-free mice with human commensal *B. fragilis*. Significantly, oral treatment with *B. fragilis* rescued the decrease in scratching bouts on days 4, 5, and 7 after treatment not only in mice pre-treated with ABX, compared to ABX group (Figure 7(b,c)), but this treatment also increased the itch threshold in H_2_O control mice (Figure 7(b,d)). Like ABX-treated mice, oral gavage of *B. fragilis* also reversed the decreased threshold of itch during observed period from day 4 to 7 after treatment in germ-free mice, relative to naïve control mice (Figure 7(e)). As expected, germ-free-induced the decrease of EST1, FTO and MRGPRF were rescued on day 7 after the gavage of *B. fragilis* (Figure 7(f)). Whereas, no locomotor impairments were observed in the treated mice (Supplemental Table S1). These results reveal the contribution of *B. fragilis* residing in the gut to changes in perceived itching in spinal FTO-dependent manner.

### Acetyl-l-carnitine ameliorates itch dysfunction in ABX mice via the gut-spinal axis

**Figure 8.**
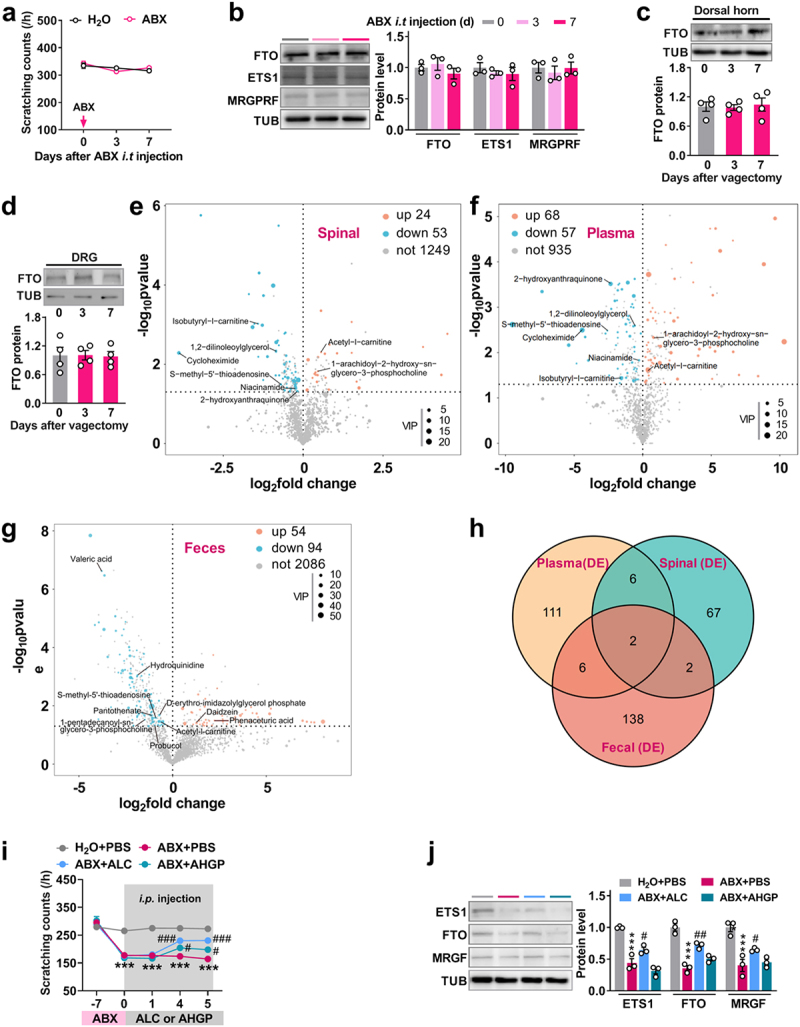
Acetyl-l-carnitine supplementation ameliorates itch dysfunction induced by the reduction of gut microbiota. a, the effect of intrathecal injection of ABX on the itch threshold in naïve mice. *n* = 6 mice/group. b, the expression level of dorsal horn FTO, EST1, and MRGPRF on days 3 and 7 after intrathecal injection of ABX in naïve mice. *n* = 3 mice/group. (c, d) FTO expression levels in the dorsal horn (c) and DRG (d) on days 3 and 7 after vagectomy surgery. One-way ANOVA, post hoc Tukey’s test. *n* = 4 mice/group. (e-g) the analysis of differential metabolites in the spinal dorsal horn (e), blood plasma (f), and feces (g) through using LC-MS/MS on day 7 after transplantation of *B. fragilis* in germ-free mice. Up, upregulated; down, downregulated; not, not changed. Spinal, dorsal spinal horn. *n* = 3 (for spinal and plasma) or 4 (for feces) mice/group. (h) the analysis of overlapping differential metabolites among the dorsal horn, plasma, and feces. DE, differential expression metabolites including up and down. (i) the time course of scratching bouts in response to chloroquine after the intraperitoneal injection of acetyl-l-carnitine (ALC) or 1-arachidoyl-2-hydroxy-sn-glycero-3-phosphocholine (AHGP) in ABX mice for continuous 5 days. **p < 0.001 versus the H_2_O + PBS group; ***p < 0.05, ^##^*P* < 0.001 versus the ABX + PBS group. Two-way ANOVA, post hoc Tukey’s test. *n* = 8 mice/group. j, the expression level of dorsal horn FTO, EST1, and MRGPRF on 5 after administration of ALC in ABX mice. *n* = 3 mice/group. **p < 0.001 versus the H_2_O + PBS group; ***p < 0.05, ^##^*P* < 0.01 versus the ABX + PBS group. One-way ANOVA, post hoc Tukey’s test. *n* = 3 mice/group.

Microbe-derived metabolites in gut are the critical mediators of the regulatory functions of nerve system tissues through escaping into the bloodstream and CNS tissues.^41^ How and which gut microbe-derived metabolites are implicated in the itch dysfunction? To rule out the possibility that antibiotics might directly affect the spinal cord, we first assessed whether direct intrathecal injection of ABX could alter itch behavior and spinal FTO expression. As expected, we found that the intrathecal injection of ABX affected neither the itch threshold (Figure 8(a)), nor the expression level of FTO, EST1 and MRGPRF in dorsal horn of naïve mice, compared to the control group (Figure 8(b)), these results exclude the direct effect of ABX on the dorsal horns. Given that vagus nerve (VN) is able to sense microbiota metabolites through its afferents and transfer information about the gut to the CNS, thus generating adaptive or maladaptive responses.^42^ To explore whether the VN participates in the itch sensation, we examined the effect of bilateral vagectomy on itch-related FTO expression in the dorsal horn. We did not observe changes in FTO expression in the dorsal horn (Figure 8(c)) or the DRG (Figure 8(d)) on day 3 or day 7 post-vagectomy, compared to the day 0 control. These findings indicate that itch-related FTO expression in the dorsal horn is independent of VN signaling.

Thus, we speculate that circulation blood may mediate the implication of gut microbiota-derived metabolites in the development of itch. To this end, we employed LC-MS/MS to analyze metabolomic changes in blood plasma, spinal cord, and feces from germ-free mice, as well as following the transplantation of *B. fragilis* into germ-free mice. The results indicated significant differences in metabolites across blood plasma, spinal cord, and feces after the transplantation of *B. fragilis* into germ-free mice, with VIP > 1 and *p* < 0.05. Specifically, there were 77 differential metabolites in the spinal cord (24 upregulated and 53 downregulated) (Figure 8(e,h)), 125 in blood plasma (68 upregulated and 57 downregulated) (Figure 8(f,h)), and 148 in feces (54 upregulated and 94 downregulated) (Figure 8(g,h)) following *B. fragilis* transplantation. Among the overlapping metabolites, six were downregulated between blood plasma and spinal cord, including 1,2-dilinoleoylglycerol, cycloheximide, isobutyryl-l-carnitine, niacinamide, S-methyl-5’-thioadenosine (SMTA), and 2-hydroxyanthraquinone; two metabolites were upregulated: acetyl-l-carnitine (ALC) and 1-arachidoyl-2-hydroxy-sn-glycero-3-phosphocholine (AHGP) (Figure 8(e,f), and Table 1). However, only two overlapping metabolites – SMTA and ALC – were found among feces, spinal cord, and plasma (Figure 8(h), and Table 2), both of which were downregulated in the feces (Figure 8(g), and Table 2).

**Table 1.** Analysis of same differential metabolites between plasma and spinal.

	Plasma			Spinal dorsal horn		
Metabolite name	Fold	P-value	VIP	Fold	P-value	VIP
2-hydroxyanthraquinone	0.19723065	0.000305271	13.00049558	0.880514412	0.048225611	3.630527375
1,2-dilinoleoylglycerol	0.670787506	0.002555902	2.015975281	0.555410327	0.004537276	1.880662533
Cycloheximide	0.046887512	0.003188225	17.91976729	0.375789953	0.00094833	1.098615939
Isobutyryl-l-carnitine	0.338056087	0.036612123	9.505462292	0.844123447	0.039871151	8.145448015
Niacinamide	0.830852196	0.014781582	2.367020615	0.067459368	0.005168741	7.205164398
S-methyl-5’ − thioadenosine	0.167455603	0.003285079	1.060609421	0.617610057	0.032257955	1.869909197
1-arachidoyl-2-hydroxy-sn-glycero-3-phospho choline	1.702065923	0.004576889	3.717759459	1.377109681	0.014933267	1.410634929
Acetyl-l-carnitine	1.321094915	0.024219028	19.40071424	1.477264931	0.00495314	1.237079309

Fold change (Fold) = (germ-free + B. fragilis)/(germ-free). VIP, variable importance in projection.

**Table 2.** Metabolites in feces overlapping with in both plasma and spinal.

Metabolite name	Fold	P-value	VIP
S-methyl-5’ − thioadenosine	0.543120702	0.026197632	2.175701854
Acetyl-l-carnitine	0.647034074	0.035092454	6.725260463

Fold change = (germ-free + B. fragilis)/(germ-free).

**Figure 9.**
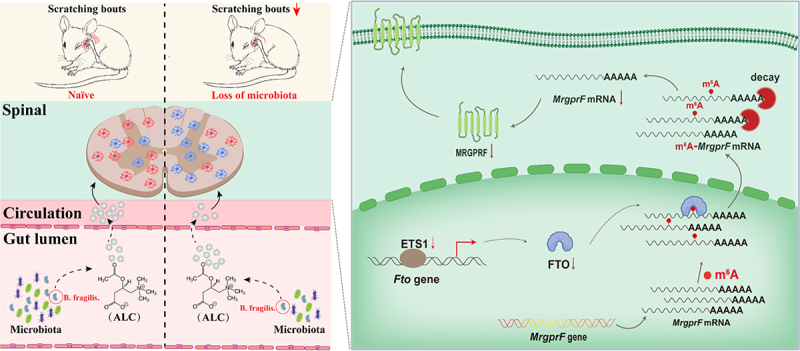
Schematic strategy for ALC’s role in improving itch dysfunction induced by deletion of gut microbiota via the gut‑spinal axis in FTO-dependent manner. Reduction of gut microbiota down-regulates FTO expression in the dorsal horn. This downregulation is attributed to a reduction in the binding of ETS1 to the F*to* promoter in dorsal horn neurons. Downregulation of FTO results in an increase in m^6^A in the *MrgprF* 3’UTR, triggering inhibition of MRGPRF protein expression and consequent genesis of itch tolerance. Treatment with *B. fragilis* can restore normal itch behavior through its derived metabolites acetyl-l-carnitine in mice with microbiota depletion.

Considering that spinal metabolites may be directly functionally associated with the regulation of itch, therefore, the upregulated ALC and AHGP in spinal dorsal and plasma were chosen as target metabolites in the following experiments. We found that the intraperitoneal injection of both ALC and AHGP elevated the decreased threshold of itch on day 4 and 5 after injection in ABX-treated mice, whereas, ALC showed the relatively effective restoration, compared with AHGP (Figure 8(i)). However, only ALC, not AHGP, was able to rescue the ABX-induced reduction of EST1, FTO, and MRGPRF in the dorsal horn of ABX mice on day 5 after administration (Figure 8(j)). These results suggest that ALC, at least in part, ameliorates the itch dysfunction induced by deletion of microbiota via the gut-spinal axis (Figure 9).

## Discussion

The gut microbiota is the largest community of microorganisms in the human body, its key role in the establishment and maintenance of health, as well as in the pathogenesis of disease, has been identified over the past two decades. Importantly, gut microbiota is integral to the development and activity of the immune and nervous systems. Compositional changes in the microbiota not only are related to intestinal diseases such as inflammatory bowel disease, gastroenteritis, and intestinal tumors,^43^ but also influence the occurrence of parenteral diseases such as cancer and nonalcoholic fatty liver disease, as well as metabolic diseases such as obesity and diabetes.^44^ Beyond this, recent studies are starting to link the gut microbiota to neurological disorders that involve neuroinflammation, such as multiple sclerosis, autism spectrum disorder, Parkinson’s disease, depression, and nociception.^45^ Interestingly, the gut microbiota has been implicated in the development of itch-induced cognitive dysfunction. Chronic itch was found to alter the composition of mouse microbiota and, furthermore, these alterations were potentially associated with dysfunction in novel object recognition.^11^ Despite this research indicating that chronic itch changes the composition profile of the gut microbiota, whether and how the converse is true – whether the gut microbiota is able to regulate itch behavior – is poorly understood. Our findings reveal that mice lacking gut microbiota displayed tolerance to pruritogens. Raising male or female mice lacking gut microbiota in the same cage as normal control mice, or fecal microbiome transplantation of normal mice, or administration with *B. fragilis* reversed the enhanced itch thresholds. These findings highlight the essential role of gut microbiota in the development and maintenance of itch and expand our understanding of how gut microbiota contributes to diverse physiological functions in the body.

To explore the potential of managing itch through gut microbiota modulation, we investigated the connections between gut microbiota and neuronal pathways, including signaling molecules, specific bacteria, and metabolites. Given the established association of spinal RNA m^6^A modification with various physiological and pathological processes, we further examined the role of spinal RNA m^6^A in itch tolerance induced by gut microbiota depletion. FTO is a demethylase specific for m^6^A sites in RNA. Our screening results revealed that depleting the gut microbiota robustly reduced *Fto* expression in the dorsal horn in a time-dependent manner, but slightly increased the expression of another demethylase, *Alkbh5*. Unexpectedly, treatment with antibiotics did not change the expression of the RNA methylases *Mettl3*, *Mettl14*, or *Wtap* in the dorsal horn. RNA m^6^A is a reversible dynamic process, but which methylase plays the functional role in RNA methylation remains a puzzle. Recent studies have found that FTO is a key regulator in the development of neurological disorders including Alzheimer’s disease,^46^ Parkinson’s disease,^19^ fragile X syndrome, and depression.^47^ A study by Li et al. confirmed the relationship between FTO and neuropathic pain.^23^ Peripheral nerve injury leads to a significant increase in FTO in the injured DRG, which contributes to the induction and maintenance of nerve-injury-induced neuropathic pain at least in part through erasing the m^6^A in *Ehmt2* mRNA and stabilizing the nerve-injury-induced *Ehmt2* mRNA/G9a increase in the injured DRG. Their data suggest that FTO may serve as a potential new target for neuropathic pain management. Itch and pain may share an epigenetic mechanism: here, we demonstrated a novel link between FTO in the dorsal horn and itch behavior. Gut microbiota depletion caused downregulation of FTO in dorsal horn and not only increased the level of spinal RNA m^6^A, but also elevated the threshold for scratching. Rescuing the downregulation of FTO restored the impaired itch threshold almost to the baseline level. Thus, FTO is a key regulatory factor in the itch tolerance induced by deletion of the gut microbiota.

Furthermore, our observations suggest that the downregulation of FTO can be attributed to decreased spinal epithelial-specific ETS1. ETS1 is a member of the ETS transcription factor family and is widely expressed in multiple tissues. It contains an evolutionarily conserved ETS domain that contributes to reorganization of the GGAA/T motif sequence in gene promoters.^48^ We found that there is an ETS1 binding motif in the *Fto* promoter. Both *in vitro* and *in vivo* results confirm the regulatory function of ETS1 in *Fto* expression. A growing body of evidence supports a strong functional connection between ETS1 and central nervous system disorders such as ischemic stroke,^49^ cerebral ischemia/reperfusion injury,^50^ multiple sclerosis,^51^ and depression.^52^ However, whether ETS1 is involved in itch was unknown. In this study, we found that ETS1 participates in gut microbiota-induced abnormal itch behavior by governing FTO expression in dorsal spinal horn. Thus, our data expand the regulatory function of ETS1 in the pathological progression of nervous system.

*Trpv1* and *MrgprF* may serve as potential targets for FTO due to the impact of gut microbiota deletion on their expression. However, for this experiment, we chose *MrgprF* as the focus for subsequent studies based on two key considerations. First, the change in *MrgprF* expression (a decrease of 47%) is more pronounced than that of *Trpv1* (a decrease of 34%) in the dorsal horn following gut microbiota deletion. Second, while *Trpv1* is a well-established gene associated with itch,^53^ the role of *MrgprF* in itch sensation remains unclear. Importantly, *MrgprF* is part of the Mas gene-related G protein-coupled receptors (MRGPRs). This family members, such as the primate-specific MRGPRX2 and its murine ortholog MRGPRB2,^54^ MRGPRA, and MRGPRX1,^55^ are reported to the responsive to various exogenous and endogenous agonists,^56^ and they are known to play a fundamental role in itch sensation.^55^ Mice lacking a cluster of *Mrgpr* genes display significant deficits in itch induced by chloroquine,^55^ but not induced by histamine. Furthermore, chloroquine can specifically activate mouse *MrgprA3* and human *MrgprX1* in dorsal root ganglia, resulting in itch responses. Activating MRGPRC11 in DRG with the specific agonist BAM8–22 induces itch in *MrgprC11* wild-type but not mutant mice. Therefore, we are particularly interested in determining whether *MrgprF* is involved in the regulation of itch sensation. However, we do not rule out the possibility that *Trpv1* may also serve as a potential downstream target of FTO, and this will be explored in future studies.

In our work, we mechanistically revealed that the essential role of FTO in the abnormal itch response induced by gut microbiota is mediated by the MRGPRF receptor in an RNA m^6^A-dependent manner. Few studies have investigated the relationship between *MrgprF* and itch behavior and the mechanism by which *MrgprF* regulates itch remains unclear. In this study, we found that gut microbiota-depletion downregulated the level of spinal *MrgprF*; this downregulation was the result of increased m^6^A, caused by the reduction in FTO in the dorsal horn. Together, our findings are the first to link gut microbiota to *MrgprF* expression via FTO control of m^6^A in *MrgprF* mRNA in the dorsal spinal horn. Our results suggest that FTO contributes to the tolerance of itch by regulating the expression of *MrgprF* in the spinal cord. Mechanistically, gut microbiota or *B. fragilis* depletion caused a decrease in RNA N^6^-methyladenosine (m^6^A) demethylase FTO expression in the dorsal horn and a consequent increase in RNA m^6^A sites in Mas-related G protein-coupled receptor F (*MrgprF*) mRNA, leading to decreased MRGPRF protein. The downregulation of FTO was triggered by inactivation of ETS proto-oncogene 1 (ETS1), a transcription factor that binds to the *Fto* promoter. Collectively, these findings underscore the critical role of gut microbiota, particularly in modulating spinal ETS1/FTO/MRGPRF, in regulating itch responses. However, further research is required to determine whether gut microbiota influences the excitability of spinal neurons mediating itch through MRGPRF or other factors.

Interactions between gut microbiota and the nervous system have been previously established. The gut microbiota can impact systems distal to the gut, including the dorsal root ganglia (DRG), spinal cord, and brain, either through the circulation of microbial metabolites or by modulating neuronal transduction via the vagus nerve. Metabolites produced by gut microbiota, such as tryptophan and lipopolysaccharides (LPS), can enter the bloodstream and reach the DRG and spinal cord.^57^ The activation of Toll-like receptors by LPS plays a crucial role in neuron – immune interactions and itch processing within the nervous system.^58^ Additionally, cytokines produced in the gut, which are significantly influenced by gut microbiota, can regulate the function of astrocytes, thereby affecting the transmission of neuronal excitability in the central nervous system.^59^ Moreover, gut microbes can modulate the concentration of metabolites or inflammatory cytokines in the intestine or bloodstream, which then circulate to the nervous system and influence neuronal excitability, thereby modulating somatic sensation.^60,61^ For example, gut microbiota depletion has been shown to prevent the development of nerve injury-induced thermal pain and inhibit glial cell activation in the spinal cord. It also ameliorates mechanical allodynia and reduces cytokine production in DRG neurons in animal models of chemotherapy-induced pain.^62^ Interestingly, metabolites produced by gut microbiota can directly reach the skin through blood circulation, disrupting skin barrier function and inducing the production of pruriceptors, which trigger skin itching, as observed in conditions such as atopic dermatitis.^63,64^ In addition to microbial metabolites, the gut microbiota can regulate the levels of certain neurotransmitters and neuromodulators, such as GABA^65^ and serotonin (5-HT),^66^ thereby influencing neuronal function. These findings deepen our understanding of the interaction between gut microbiota and the nervous system. Our study further explores how gut microbiota may interact with itch processing through the FTO-mediated RNA-m^6^A-dependent pathway in the spinal cord. However, it remains unclear whether and how spinal glial cell activation, cytokines, neurotransmitters, or neuromodulators are involved in the gut microbiota-modulated FTO signaling pathway in itch processing.

Another significant finding in this study is that partial restoration of gut microbiota can reverse itch behavior following ABX treatment. Among the microbiota, *B. fragilis* may play a crucial role in the development of itch sensation. *B. fragilis* is a major species of *Bacteroidota*, which are a highly abundant Gram-negative bacterial phylum in the human gastrointestinal tract. *B. fragilis* is about 100-fold more abundant than *Escherichia coli* and secretes several regulatory factors into host intestinal cells or the blood to modulate CNS diseases.^8^ For example, polysaccharide A (PSA) produced by *B. fragilis* has been identified as a molecule that elicits beneficial immune responses for both commensals and host.^67^ Oral treatment with *B. fragilis* increases PSA levels and protects against CNS demyelination and inflammation in a mouse model of autoimmune encephalomyelitis via Toll-like receptor 2.^40^ As well as *B. fragilis* produces sphingolipids ^68^ and polysaccharides^69^ into the circulation, which plays important roles in neuronal myelination, neuroinflammation,^70^ neuronal excitability^69^[29], chronic pain,^71^ Alzheimer’s disease,^72^ and autism spectrum disorder.^8^ Our data indicate that *B. fragilis* may have a therapeutic effect in dysfunctional itch.

Moreover, in addition to *B. fragilis*, other species within the *Bacteroides* genus may also play significant roles in sensory modulation. For instance, *B. acidifaciens* has been demonstrated to produce acetate, which can activate hypothalamic neurons and modulate energy homeostasis, highlighting its potential role in gut-brain axis signaling.^73^ However, current research predominantly focuses on the genus level rather than the species level, highlighting the need to further investigate the specific functions of individual *Bacteroides* species in future studies. Studies have shown that dysbiosis of *Bacteroides* and related metabolic activities may be linked to the pathogenesis of central nervous system disorders, such as autism spectrum disorder (ASD).^74^ The reduction of *Bacteroides* has been associated with altered tryptophan metabolism, which plays a role in modulating mood and behavior through the microbiota-gut-brain axis.^75^ Therefore, understanding the specific contributions of *Bacteroides* species to sensory modulation and exploring their therapeutic potential in managing sensory-related disorders should be a key focus of future research.

Considering that gut microbiota-derived metabolites are key hubs connecting the gut microbiome and physiological and pathological progression of host, primarily by regulating key signaling pathways in cells and multiple immune cells.^76^ Gut microbiota-derived metabolites are detectable in a wide range of biological tissues, including serum, feces, urine, liver and cerebrospinal fluid.^77,78^ The profile of metabolites exhibits a dramatic alteration in germ-free mice, compared with conventional mice, including such several tissues as urine, liver, kidney and gut tissues.^78,79^ Antibiotics can alter these composition of the metabolites in rodents.^80^ For example, indole-3-propionic acid (IPA), a microbial metabolite, is observed to be absent in germ-free mice, but is reintroduced following monocolonization with *Clostridium sporogenes*.^79,81^ In this study, given that *B. fragilis* at least partly contributes to the restoration of the itch dysfunction, we investigate which metabolites is potentially derived by *B. fragilis*, therefore, we analyzed the changes of metabolites in both plasma and dorsal spinal horn after reintroducing *B. fragilis* in the germ-free mice. Acetyl-l-carnitine (ALC) exhibited the significant increasement in both plasma and dorsal spinal horn, the administration of ALC in ABX mice improved itch dysfunction in FTO-dependent manner. It appears that *B. fragilis* contributes to itch sensation by modulating ALC levels in the dorsal spinal horn.

However, one unexpected finding was the significant decrease in ALC levels in the feces following the administration of *B. fragilis* in the germ-free mice. Consequently, although we identified that ALC is derived from the gut microbiota, we cannot determine whether it is exclusively produced by the microbiota, derived from both the host and the microbiota, or sourced from both the microbiota and dietary components. In fact, the metabolites study of host-microbiota interactions remains challenging due to the high degree of crosstalk within and between kingdoms.^76^ For example, a recent report indicates that some metabolites exhibit opposite trends in plasma and feces. Furthermore, metabolome profiling of plasma, feces, and urine revealed that, among these microbiota-derived 11 metabolites, two were completely derived from the microbiota, one was derived from both the host and the microbiota, and one was derived from both the microbiota and dietary component.^82^ Thus, future research is needed to investigate the precise source of ALC in the spinal cord.

ALC is acyl-derivatives of free carnitine, the gut- and blood-brain barrier absorb ALC easier than carnitine. ALC is described as a drug agonist of mitochondrial function, a neuronal growth factor and with an antioxidant effect on CNS neurons.^83^ ALC has multiple neurobiological effects which may possibly be beneficial in degenerative brain disease. The previous report showed that ALC prolongs survival of cultured rat brain cells after exposure to neurotoxic stimuli^84^ and improved cognitive functioning in rats.^85^ Furthermore, endogenous ALC has been demonstrated to modulate mitochondrial function via the epigenetic pathway, and is associated with ischemia,^86^ autism,^87^ stress,^88^ depression and Alzheimer’s Disease.^89^ Here, we firstly support the evidence that ALC participates in modulation of itch behavior, this expands the functions of ALC in physiological and pathological progression. Our findings suggest that the reduction of *B. fragilis* induced by ABX treatment leads to an increase in ALC levels in the spinal cord, which further suppresses the expression of spinal MRGPRF through the ETS1/FTO pathway, resulting in weakened transmission of itch signals and reduced itch perception. However, the precise mechanisms linking ALC to ETS1/FTO/MRGPRF and how their interactions influence the excitability of spinal neurons require further investigation in future studies.

In summary, we established a connection between gut microbiota and itch. Mechanistically, gut microbiota deletion decreases the ETS1 level, which triggers a reduction in FTO expression and consequent elevation of *MrgprF* m^6^A in dorsal spinal horn, resulting in a decrease in *MrgprF* expression and increased tolerance to itch. Oral gavage with *B. fragilis* or the intraperitoneal administration of acetyl-l-carnitine, in part, corrected the ABX-induced itch dysfunction via FTO-dependent manner. This is the first description of the involvement of RNA m^6^A modification in gut microbiota-induced itch behavior. Our findings support a gut microbiome – spinal connection in a mouse model of itch and may provide a new therapeutic avenue in the treatment of itch.

## Supplementary Material

Supplemental Material

## Data Availability

The data from this study are available from the corresponding author upon reasonable request.
