# Supplementary material for: Gut microbes–spinal connection is required for itch sensation
Source: Gut Microbes. 2025 Apr 27;17(1):2495859. doi: 10.1080/19490976.2025.2495859 (PMC12036491; doi:10.1080/19490976.2025.2495859)
Supplement: Supplemental Material [file KGMI_A_2495859_SM2297.docx]

**Supplemental Tables**

**Table S1.** Test of locomotor function post-injection of manipulation tools.

| Treatment groups | Placing | Grasping | Righting |  |
| --- | --- | --- | --- | --- |
| H_2_O | 8(0) | 8(0) | 8(0) |  |
| ABX | 8(0) | 8(0) | 8(0) |  |
| Scrambled siRNA | 8(0) | 8(0) | 8(0) |  |
| si-Ets-94 | 8(0) | 8(0) | 8(0) |  |
| H_2_O + pCDH-Gfp | 8(0) | 8(0) | 8(0) |  |
| H_2_O + pCDH-Ets1 | 8(0) | 8(0) | 8(0) |  |
| ABX + pCDH-Gfp | 8(0) | 8(0) | 8(0) |  |
| ABX + pCDH-Ets1 | 8(0) | 8(0) | 8(0) |  |
| H_2_O + pCDH-Fto | 8(0) | 8(0) | 8(0) |  |
| ABX + pCDH-Fto | 8(0) | 8(0) | 8(0) |  |
| si-Fto-403 | 8(0) | 8(0) | 8(0) |  |
| si-Fto-66 | 8(0) | 8(0) | 8(0) |  |
| PLV-Scr-shRNA  PLV-Fto-shRNA | 8(0)  8(0) | 8(0)  8(0) | 8(0)  8(0) |  |
| CRISPR-dCasRx + Scrambled gRNA | 8(0) | 8(0) | 8(0) |  |
| CRISPR-dCasRx + gRNA-611 | 8(0) | 8(0) | 8(0) |  |
| ABX + CRISPR-dCasRx + Scr gRNA | 8(0) | 8(0) | 8(0) |  |
| ABX + CRISPR-dCasRx + gRNA-611 | 8(0) | 8(0) | 8(0) |  |
| ABX + CRISPR-dCasRx/Fto + Scr gRNA | 8(0) | 8(0) | 8(0) |  |
| ABX + CRISPR-dCasRx/Fto + gRNA-611 | 8(0) | 8(0) | 8(0) |  |
| ABX+CRISPR-dCasRx/Mettl3+Scr gRNA | 6(0) | 6(0) | 6(0) |  |
| ABX+CRISPR-dCasRx/Mettl3+gRNA-611 | 8(0) | 8(0) | 8(0) |  |
| H_2_O + pCDH-MrgprF | 8(0) | 8(0) | 8(0) |  |
| ABX + pCDH-MrgprF | 8(0) | 8(0) | 8(0) |  |
| pCDH-MrgprF + si-Fto | 8(0) | 8(0) | 8(0) |  |
| si-MrgprF-524 | 8(0) | 8(0) | 8(0) |  |
| si-MrgprF-857 | 8(0) | 8(0) | 8(0) |  |
| H_2_O + *B. fragilis* | 8(0) | 8(0) | 8(0) |  |
| ABX + *B. fragilis* | 8(0) | 8(0) | 8(0) |  |

Data are mean (SEM). n=6-8/group; five trials. No significance; one-way

ANOVA (response time vs treated groups) followed by post hoc Tukey test.

**Table S2.** All primers and probes used in this study.

| Name | Sequences |
| --- | --- |
| **RT-qPCR** | |
| Fto-F | 5′-TTCATGCTGGATGACCTCAATG-3′ |
| Fto-R | 5′-GCCAACTGACAGCGTTCTAAG-3′ |
| Mettl3-F | 5′-CTGGGCACTTGGATTTAAGGAA-3′ |
| Mettl3-R | 5′-TGAGAGGTGGTGTAGCAACTT-3′ |
| Mettl14-F | 5′-CTGAGAGTGCGGATAGCATTG-3′ |
| Mettl14-R | 5′-GAGCAGATG TATCATAGGAAGCC-3′ |
| Wtap-F | 5′-TAGACCCAGCGATCAACTTGT-3 ′ |
| Wtap-R | 5′-CCTGTTTGGCTATCAGGCGTA-3′ |
| Alkbh5-F | 5′-CGCGGTCATCAACGACTACC-3′ |
| Alkbh5-R | 5′-ATGGGCTTGAACTGGAACTTG-3′ |
| E2f4-F | 5′-CTCACCACCAAGTTCGTGTC-3′ |
| E2f4-R | 5′-TCTCGATCAGACCGATGCCTT-3′ |
| E2f6-F | 5′-GATGGCATCGAACTGGTGGAA-3′ |
| E2f6-R | 5′-CCCCAAAGTTGTTCAGGTCAG-3′ |
| Ets1-F  Ets1-R  Tcf12-F  Tcf12-R  Zic2-F  Zic2-R  Bcl6-F  Bcl6-R  Etv4-F  Etv4-R  MrgprbF | 5′-ACAGACTACTTTGCCATCAAGCA-3′  5′- ACGCTCTCAAAAGAGTCCTGG-3′  5′-CGCGATAGGGACCGACAAG-3′  5′-ACCCGCTGAACTGACTACTTC-3′  5′-CAAGGTCCGGGTGCTTACC-3′  5′-ATTAAAGGGAGGCCCCGAATA-3′  5′-CCGGCACGCTAGTGATGTT-3′  5′-TGTCTTATGGGCTCTAAACTGCT-3′  5′-CATTCCCAGATGATGTCTGCAT-3′  5′-CCACAGTTGTAAGGCACCCC-3′  5′-AGCCCTGCTGAGGAGACGGT-3′ |
| MrgprbR | 5′-TGTCGTACATAGTGCTCAGAT-3′ |
| MrgpreF | 5′-GGAGAAATGGCTTTCAACCTGA-3′ |
| MrgpreR | 5′-GAGAAGGGGTTCCTGTAGACA-3′ |
| MrgprfF | 5′-ATGGCCGGAAACTGTTCATGG-3′ |
| MrgprfR  Trpv1F | 5′-TGGTCAGAAATCCTCTGCTGTA-3′  5′- CCCGGAAGACAGATAGCCTGA-3′ |
| Trpv1R | 5′- TTCAATGGCAATGTGTAATGCTG-3′ |
| Trpm4F  Trpm4R | 5′- GAAACGCACGTCTCAGAAGGT-3′  5′-CAGCCATCCAAGTCAGGACTC-3′ |
| Mouse-Gapdh-F | 5′-ACCACAGTCCATGCCATCAC-3′ |
| Mouse-Gapdh-R | 5′-TCCACCACCCTGTTGCTGTA-3′ |
| **siRNA** | |
| Fto-siRNA-66-S | 5′-GCUUGAAGACACUUGGCUUTT-3′ |
| Fto-siRNA-66-AS | 5′-AAGCCAAGUGUCUUCAAGCTT-3′ |
| Fto-siRNA-403-S | 5′-GCAUGUCAGACCUUCCUAATT-3′ |
| Fto-siRNA-403-AS | 5′-UUAGGAAGGUCUGACAUGCTT-3′ |
| Ets1-siRNA-94-S | 5′-GCAGAUGUCCCGCUGUUAATT-3 |
| Ets1-siRNA-94-AS | 5′-UUAACAGCGGGACAUCUGCTT-3′ |
| Ets1-siRNA-396-S | 5′-GCAGAAAGAGGAUGUGAAATT-3′ |
| Ets1-siRNA-396-AS | 5′-UUUCACAUCCUCUUUCUGCTT-3′ |
| MrgprF-siRNA-524-S | 5′-CCAGUAUCCACAAUUACUUTT-3′ |
| MrgprF-siRNA-524-AS | 5′-AAGUAAUUGUGGAUACUGGTT-3′ |
| MrgprF-siRNA-857-S | 5′-CCAAACCCAUCGUCUACUUTT-3′ |
| MrgprF-siRNA-857-AS | 5′-AAGUAGACGAUGGGUUUGGTT-3′ |
| Scrambled-siRNA-S | 5′-UUCUCCGAACGUGUCACGUTT-3′ |
| Scrambled-siRNA-AS | 5′-ACGUGACACGUUCGGAGAATT-3′ |
| **RIP-PCR primers for MrgprF m^6^A** | |
| a-m^6^A-F12 5′-TGGTACAGTCTGCCGCAACA-3′ | |
| a-m^6^A-R1 5′-AGTACCATGAGTGGGCAGAA-3′  a-m^6^A-R2 5′-TCGGCACTCTACGTGCAGGAT-3′  (“a” indicate the first m^6^A sites, F12-R1 for “1” primer pair, F12-R2 for “2” primer pair, the following labels are alike ) | |
| b-m^6^A-F1 5′-TGCCAGGGAGAAGACCTGC-3′ | |
| b-m^6^A-F2 5′-AGGCTGAGCAGCCACCTCT-3′ | |
| b-m^6^A-R12 5′-CAACTTTGGTACACTGAGTCA-3′ | |
| c-m^6^A-F12 5′- ACTGTAGTCCACTGCTAAGTC-3′ | |
| c-m^6^A-R1 | 5′-ATGATGAGTGACATCGTGGT-3′ |
| c-m^6^A-R2 | 5′-ACAGGCCACTGTGCTGGATG-3′ |
| **ChIP-PCR for Fto promoter** | |
| ChIP-Fto-F | 5′-AGCCTTTGTCATTATCCCAGA-3′ |
| ChIP-Fto-R | 5′-CAGATAGCAGAAGAGGAACTAT-3′ |
| **gRNA vector targeting MrgprF in CRISPR-CasRx** | |
| pLH-sgRNA-490F | 5′-accgAGGCCAGATCCCATCCCATTA-3′ |
| pLH-sgRNA-490R | 5′-aaacTAATGGGATGGGATCTGGCCT-3′ |
| pLH-sgRNA-611F | 5′-accgGGAAGATGCTGAGTGCAGTCA-3′ |
| pLH-sgRNA-611R | 5′-aaacTGACTGCACTCAGCATCTTCC-3′ |
| pLH-sgRNA-Scramble-F | 5′-accgTTCTCCGAACGTGTCACGT-3′ |
| pLH-sgRNA-Scramble-R | 5′-aaacACGTGACACGTTCGGAGAA-3′ |
| **Full-length expression vector or CRISPR-dCasRx/Fto or CRISPR-dCasRx/Mettl3 vector or shRNA** | |
| pCDH-Fto-F | 5′-AATTCGAATTTAAATCGGATCCATGAAGCGCGTCCAGACCGC-3′ |
| pCDH-Fto-R | 5′-GATCCTTGCGGCCGCGGATCCCTAGGATCTTGCTTCCAGCA -3′ |
| pCDH-Ets1-F | 5′-AATTCGAATTTAAATCGGATCCATGAAGGCGGCCGTCGATC-3′ |
| pCDH-Ets1-R | 5′-GATCCTTGCGGCCGCGGATCCCTAGTCAGCATCCGGCTTTAC-3′ |
| pCDH-MrgprF-F | 5′-AATTCGAATTTAAATCGGATCCATGGCCGGAAACTGTTCATG-3′ |
| pCDH-MrgprF-R | 5′-GATCCTTGCGGCCGCGGATCCTCAGGATGCGTTCCCAGAGG-3′ |
| dCasRx/Fto-F | 5′- gtgtccggcaattccggatccATGAAGCGCGTCCAGACCGC-3′ |
| dCasRx/Fto-R | 5′- ctttttcttaggtccggatccCTAGGATCTTGCTTCCAGCA-3′ |
| dCasRx/Mettl3-F | 5′- gtgtccggcaattccggatccATGTCGGACACGTGGAGC-3′ |
| dCasRx/Mettl3-R | 5′-ctttttcttaggtccggatccCCTATAAATTCTTAGGTTTAG-3′ |
| PLV-shR-Fto-F | 5′-CGCGTGCATGTCAGACCTTCCTAATTCAAGAGATTAGGAAGGTCTGAC  ATGCTTTTTGGAAAT-3′ |
| PLV-shR-Fto-R | 5′-CGATTTCCAAAAAGCATGTCAGACCTTCCTAATCTCTTGAATTAGGAA  GGTCTGACATGCA-3′ |
| **Luciferase reporter vector** | |
| pGL6-Fto-promoter-F | 5′-CCGGTACCGCTAGCCTCGAGAGCCTTTGTCATTATCCCAGA-3′ |
| pGL6-Fto-promoter-R | 5′-CTACGCGTGAGCTCCTCGAGCTGGACCCCGCCTCCTGTGT-3′ |

RT: Reverse-transcription. F: Forward. R: Reverse. S, sense. AS: antisense

**Supplemental Figures**

**Figure S1**

**
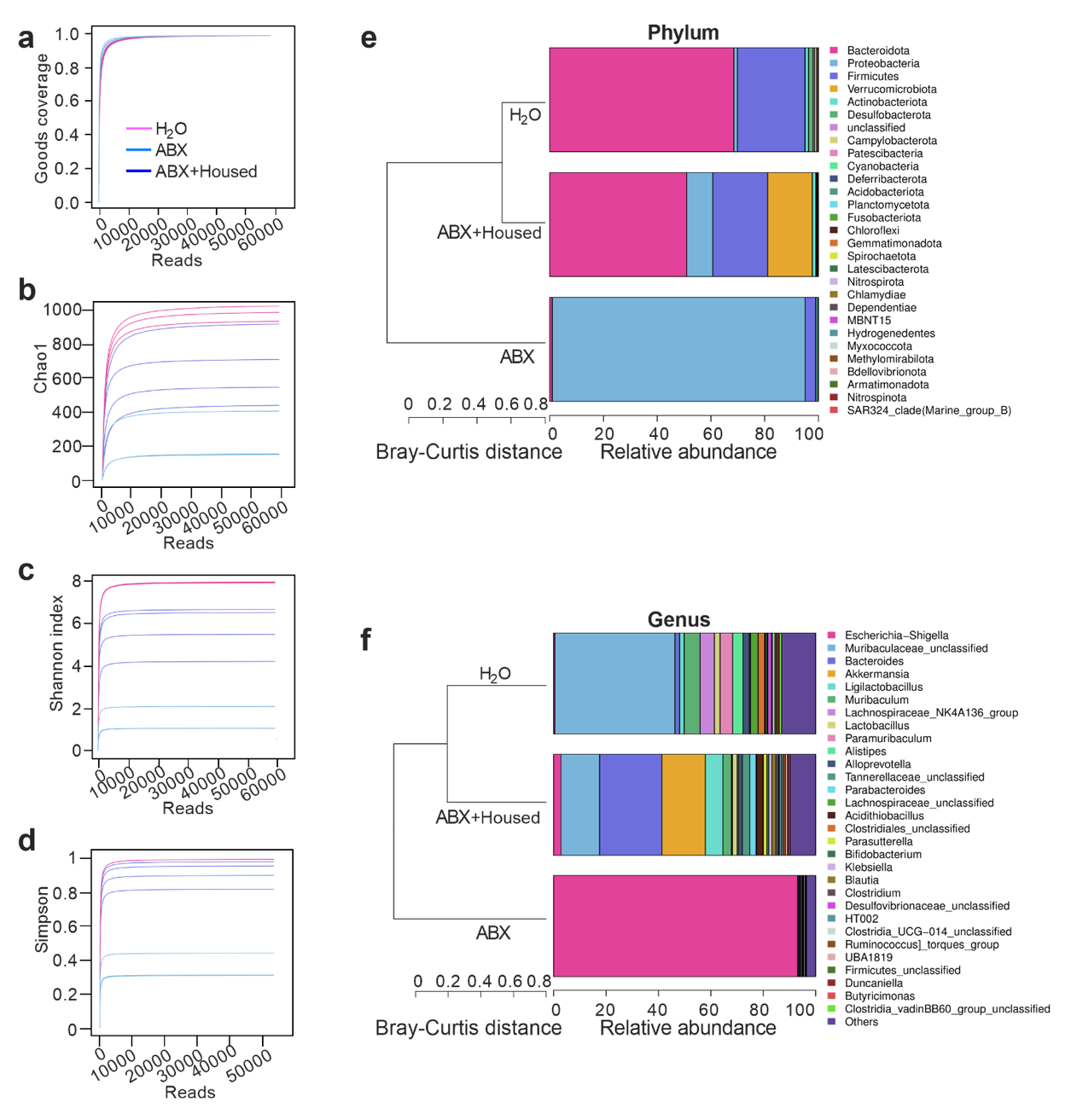
**

**Figure S2**


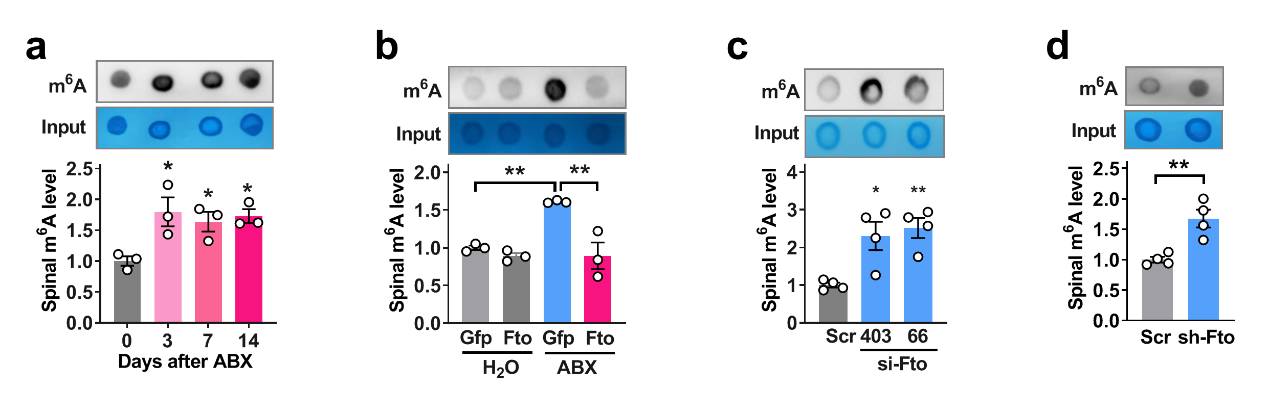


**Figure S3**


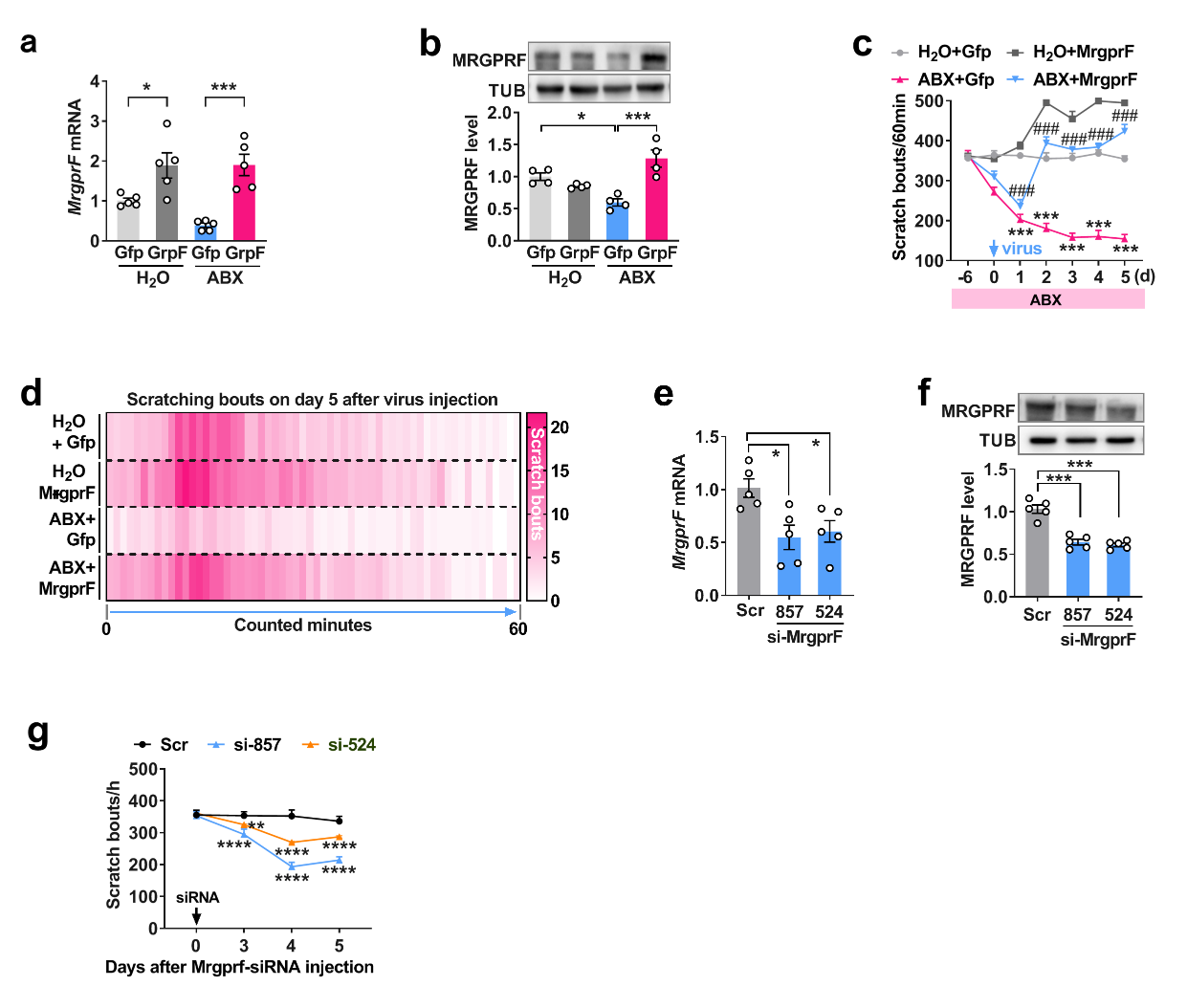


**Supplemental Figure Legends**

**Fig. S1 Oral administration with antibiotics (ABX) changes the** **constitution of gut microbiota. a,** Good coverage index for the 16S rDNA sequencing samples. **b, c**, Alpha diversity analysis shows the Chao1 (b), and Shannon (c) and simpson (d) indices of fecal 16S rDNA sequencing data from mice after 7 days drinking water (control) or water with ABX. n = 3 repeats. **e, f,** Bar-plot analysis showing the Phylum-level (e) and Genus-level (f) comparison of proportional microbiota abundance in fecal samples from ABX and control (H_2_O) mice. Each row is from one mouse sample.

**Fig. S2 Downregulation of FTO is responsible for the increase in global spinal m^6^A in mice with gut-microbiota depletion. a,** The level of m^6^A in the dorsal horn after ABX treatment. *P < 0.05, **P < 0.01 versus day 0, one-way ANOVA, post hoc Tukey’s tests. n = 3 mice/group. **b,** The level of m^6^A in the dorsal horn in mice on day 5 after intrathecal injection of Lenti-*Fto* in mice treated with ABX for 7 days. *P < 0.05, **P < 0.01 versus corresponding groups, one-way ANOVA, post hoc Tukey’s tests. n = 3 mice/group. **c,** Dorsal horn m^6^A levels on day 2 after intrathecal injection of *Fto* siRNA-403 or -66 in naïve mice. **P < 0.01 versus Scr, one-way ANOVA, post hoc Tukey’s tests, n = 4 mice/group. **d,** The level of dorsal horn m^6^A after intrathecal injection of Lenti-*Fto*-shRNA in naïve mice. **P < 0.01 versus scrambled shRNA, two-tailed unpaired Student’s t test, n = 4 mice/group.

**Fig. S3 *MrgprF* participates in modulation of itch behavior. a, b,** The expression of *MrgprF* mRNA (a) and protein (b) in the dorsal horn on day 5 after intrathecal injection of Lenti-*MrgprF* (GrF) or Lenti-*Gfp* (Gfp). **P < 0.01, ***P < 0.001 versus corresponding groups, one-way ANOVA, post hoc Tukey’s tests. n = 4-5 mice/group. **c, d,** The time course (c) and heat map on day 5 (d) of scratching bouts to chloroquine stimulus after intrathecal injection of Lenti-*MrgprF* (MrgprF) or Lenti-*Gfp* (Gfp) in ABX mice. *P < 0.05, **P < 0.01 versus H_2_O+Gfp; ##P < 0.01 versus ABX+Gfp. N = 6 mice/group. **e, f,** The level of *MrgprF* mRNA (e) and protein (f) in the dorsal horn on day 2 after intrathecal injection of *MrgprF* siRNA (si-*MrgprF*)-857 (CDS: 857-875) and -524 (CDS: 524-542, the first nucleotide of CDS designated as +1) in naïve mice. *P < 0.05, **P < 0.01 versus Scr, one-way ANOVA, post hoc Tukey’s tests. n = 5 mice/group. **g,** The time-course analysis of scratching bouts to chloroquine stimulus after injection of si-*MrgprF*-857 (g) and -524 (h) in naïve mice. **P < 0.01, ****P < 0.0001 versus Scr group at the corresponding time, two-way ANOVA, post hoc Tukey’s tests. n = 5 mice/group.
